# A Computational Model of Neuro-Glio-Vascular Loop Interactions

**DOI:** 10.1371/journal.pone.0048802

**Published:** 2012-11-20

**Authors:** Bankim Subhash Chander, V. Srinivasa Chakravarthy

**Affiliations:** Department of Biotechnology, Indian Institute of Technology, Madras, Chennai, India; Univ. Kentucky, United States of America

## Abstract

We present a computational, biophysical model of neuron-astrocyte-vessel interaction. Unlike other cells, neurons convey “hunger” signals to the vascular network via an intervening layer of glial cells (astrocytes); vessels dilate and release glucose which fuels neuronal firing. Existing computational models focus on only parts of this loop (neuron→astrocyte→vessel→neuron), whereas the proposed model describes the entire loop. Neuronal firing causes release of a neurotransmitter like glutamate which triggers release of vasodilator by astrocytes via a cascade of biochemical events. Vasodilators released from astrocytic endfeet cause blood vessels to dilate and release glucose into the interstitium, part of which is taken up by the astrocyticendfeet. Glucose is converted into lactate in the astrocyte and transported into the neuron. Glucose from the interstitium and lactate (produced from glucose) influx from astrocyte are converted into *ATP* in the neuron. Neuronal *ATP* is used to drive the Na^+^/K^+^ATPase pumps, which maintain ionic gradients necessary for neuronal firing. When placed in the metabolic loop, the neuron exhibits sustained firing only when the stimulation current is more than a minimum threshold. For various combinations of initial neuronal [*ATP*] and external current, the neuron exhibits a variety of firing patterns including sustained firing, firing after an initial pause, burst firing etc. Neurovascular interactions under conditions of constricted vessels are also studied. Most models of cerebral circulation describe neurovascular interactions exclusively in the “forward” neuron→vessel direction. The proposed model indicates possibility of “reverse” influence also, with vasomotion rhythms influencing neural firing patterns. Another idea that emerges out of the proposed work is that brain's computations may be more comprehensively understood in terms of neuro-glial-vascular dynamics and not in terms of neural firing alone.

## Introduction

**Figure 1:**
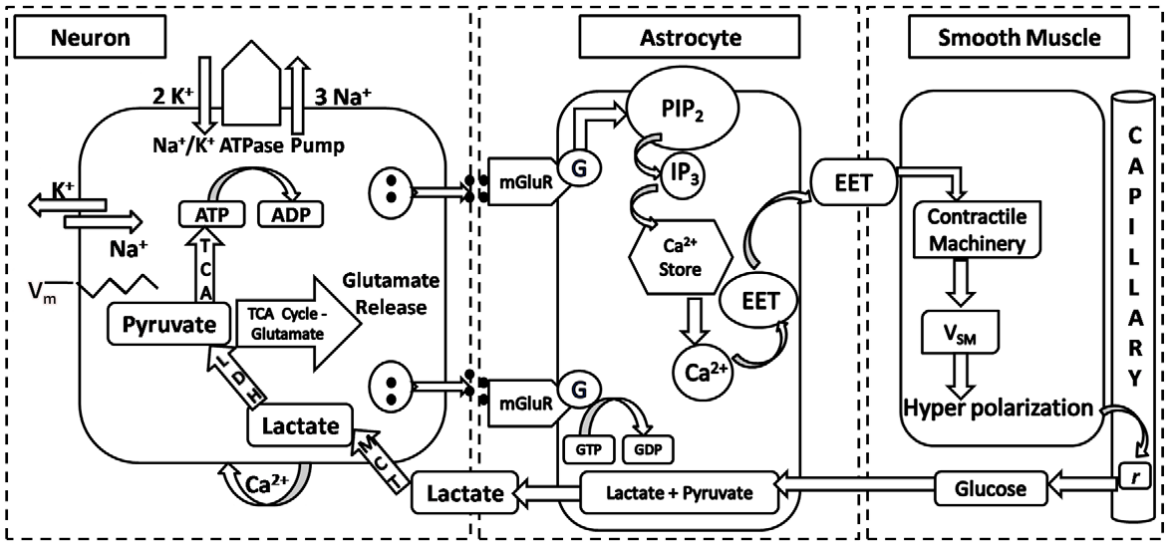
Schematic of biochemical signaling involved in neuron→astrocyte→vessel→neuron coupling.

Active neurons employ various biochemical signaling mechanisms to regulate local blood flow, some of which are astrocyte-mediated. Astrocytic processes, expressing specific receptors for neurotransmitters, surround synapse and thus can be stimulated by release of neuronal neurotransmitter in the synaptic cleft [1]. Astrocytic endfeet also envelope blood vessels and, in response to release of neurotransmitters in synapse, release vasoactive molecules to control vessel diameter [2]; [3]. Vasodilation increases blood flow and improves oxygen and glucose delivery to active neuronal tissue, both via an astrocyte-mediated pathway and also directly by release into the interstitium. Astrocyte-mediated pathways facilitate glucose uptake from astrocytic endfeet which is metabolized to pyruvate and lactate in the astrocyte [1]. Neurons uptake lactate released by astrocytes into the interstitium via specific lactate transporters to produce Adenosine Triphosphate (*ATP*) molecules [1]. *ATP* is used to fuel the pump activity necessary to restore ionic gradients necessary to sustain neuronal activity. In the present work, the above mentioned events in neuro-glial-vascular interaction have been modeled in an elaborate biophysical model (fig. 1).

### 1.1. Neuronal Signaling

Action potential propagation through neuron disturbs ionic (*Na^+^* and *K^+^*) gradients required to sustain neuronal firing. The efflux of *K^+^* and influx of *Na^+^* during action potential propagation is reversed by Na^+^/K^+^ ATPase pump at the expense of *ATP*. Neuron conveys its “hunger signals”, both by direct action on the vessels by release of vasodilators like NO [4], and indirectly via astrocyte-mediated pathways. On arrival of action potential at the synapse the neuron releases neurotransmitter (glutamate) into the synapse which activates neuronal *NMDA* (N-methyl- D-aspartate) receptors leading to activation of neuronal nitric oxide synthase (*nNOS*) and subsequent catalysis of *NO* production [2]. *NO* diffuses across neuronal tissue and acts on smooth muscles to cause vasodilation [5]. The mechanism by which NO acts on smooth muscle cells to cause vasodilation is not well understood and hence we have not considered this biophysical pathway in the proposed model. Also, glutamate release into the synapse by neuron facilitates astrocyte-mediated vasodilation. Astrocytic processes engulfing the synapse are found to express a plethora of metabotropic receptors which are activated by glutamate, norepinephrine, *GABA*, acetylcholine, histamine, adenosine and *ATP* [2]. Activation of these metabotropic receptors triggers cytosolic *Ca^2+^* oscillations in astrocytes which may be transmitted to neighboring astrocytes via gap junctions to elicit astrocyte-mediated vasodilation.

### 1.2. Astrocyte-Mediated Vasodilation

Activation of metabotropic glutamate receptors (*mGluR*) on astrocyticprocesses by synaptic glutamate triggers a cascade of biochemical reactions leading to release of vasoactive molecules. Activation of *mGluR* increases cytosolic [*Ca^2+^*] [6] which consequently activates phospholipase *A_2_* evoking production of arachidonic acid (*AA*) from membrane phospholipids. *AA* is precursor to various vasoactive molecules, including prostaglandins and epoxyeicosatrienoic acids (*EETs*) [7]. Astrocytic processes which engulf the synapse also uptake glutamate via specific glutamate transporters (*EAAT* 1 and 2). Glutamate is co-transported with *Na^+^* resulting in activation of Na^+^/K^+^ ATPase pump. Elevation of [*Ca^2+^*]_i_ leads to opening of large conductance *Ca^2+^*-activated *K^+^* (*BK*) channels in astrocyte. *BK* channels allow release of *K^+^* ions from astrocyticendfeet which also compensates for *K^+^* uptake by Na^+^/K^+^ ATPase pump [7]. Hyperpolarization of vascular smooth muscle which causes vascular dilation is due to increase in conductance of smooth muscle Ca^2+^-activated *K^+^* channels(Koehler et. al., 2006). Opening of Ca^2+^ activated *K^+^* channels makes the membrane potential more negative and consequently blocks the L-type voltage-gated *Ca^2+^* channels [8]. This reduces [*Ca^2+^*]_i_ and consequently suppresses phosphorylation of myosin light chain which is essential for maintaining contracted state of smooth muscle.

### 1.3. Metabolic Feedback

Consumption of *ATP* by *Na* ^+^/*K* ^+^ ATPase pump stimulates glucose uptake and glycolysis in neuron. Hence release of glutamate in synapse triggers activity-dependent glucose uptake and glycolysis. Monocarboxylate transporters (*MCT 1 and2*) shuttle lactate to neuron (Magistretti & Pellerin, 1999) via interstitium which is converted to pyruvate by lactate dehydrogenase (*LDH*) enzyme. Pyruvate is metabolized via tricarboxylic acid (*TCA*) cycle to produce *ATP* both in neuron and astrocyte [9]. *ATP* produced in astrocyte is used for sustaining housekeeping functions and buffering of *K* ^+^ ions. *Na* ^+^/*K* ^+^ ATPase pump is fueled by astrocytic *ATP* to buffer *K* ^+^ [10] ions and recycle *Na* ^+^ ions co-transported into the astrocyte along with glutamate molecules. *Na* ^+^/*K* ^+^ ATPase pump in neuron actively uptakes *K* ^+^ ions and removes *Na* ^+^ ions to restore the ionic gradients lost during action potential propagation.

From the above discussion it is clear that neurons, astrocytes and vessels form a closed loop information transfer system and astrocytes can be visualized as a hub, relaying biochemical signals between neurons and vessels in the forward direction, and conveying energy molecules from the vessels to the neurons in the reverse direction. To the best of our knowledge there is no existing model to simulate the entire information transfer system.

Although a model of neuron-astrocyte-vessel system has an obvious interest from basic science point of view, such a model also has an applied dimension, particularly in the area of functional neuroimaging [5]. Various biomedical imaging techniques like Functional Magnetic Resonance Imaging (*f*MRI) and Positron Emission Tomography (PET) measure hemodynamic response as an indicator of neuronal activity [11]; [12]. Hemodynamic response is often conceptualized as a unidirectional influence, arising from neurons and acting on vessels. Even models that seek to infuse quantitative rigor in our understanding of neurovascular interactions typically capture only the forward pathway (neuron→astrocyte→vessel) and ignore the effect of metabolic feedback on neural activity [13]. Therefore, it is essential to study the effect of metabolic feedback on neuronal activity to bridge the gap between measured hemodynamic response and ongoing neural activity.

Glial function is often described as if it is under passive control of neural signals. However, low-frequency spontaneous Ca^2+^ oscillations were discovered in glial cells [14]; these oscillations were shown to cause NMDA-receptor-dependent excitation and calcium transients in neighboring neurons [15]. Furthermore, calcium changes in astrocytes were shown to result in Ca^2+^ oscillations in myocytes of parenchymal arterioles in brain slices [16]. Similarly small vessels are known to possess spontaneous oscillations known as vasomotion [17]. These biological data urge to move away from a picture of neuro-glio-vascular interaction in which glial and vascular networks are under total control of neural activity, and adopt a perspective in which neurons, astrocytes, and vessels are semi-independent networks operating in tandem.

### 1.4. Existing Models

Various models have been proposed to describe various components of neuro-glial-vessel interactions. Kager et al. (2000) [10] presented a model of a neuron in which exchange of ions between the cytoplasm and the interstitium is described. The model also includes glial buffering of *K* ^+^ in the interstitium. Ion exchange between neuron and interstitium described in the proposed model is designed on the lines of [10]. Nadkarni and Jung (2003, 2004) [18], [19] proposed a minimal model of neuron-astrocyte interaction. This model describes the possible role of astrocytes in modulating ongoing neuronal activity; but the model has no reference to vascular coupling. Another model of neuroglial interaction, proposed by Postnov et al. (2007) [20], incorporates subunits of tripartite synapse which include presynaptic neuron, synaptic terminal, postsynaptic neuron and a glial cell. The model also predicts that glial feedback can influence long-term potentiation of the synapse. Gibson et al. (2007) [5] and Bennett et al.(2008) [3] presented a biophysical model that describes the chain of events from increased neural activity to local changes in CBF; astrocytes play a crucial role in this model. This model captures astrocytic *IP_3_*-mediated release of *EET* which is a potent vasodilator. In the cerebrovascular network model of Boas et al. (2008) [13], the relation between cerebral metabolic rate and local changes CBF is expressed. However, the model has no explicit representation of neural orglial cells. An abstract network model of neuro-glial-vascular interaction by Gandrakota et al. (2010) [21] proposed a theory of the need for a large glial layer in cerebrovascular circulation. However, the model involved networks of abstract nonlinear oscillators and is not biophysically grounded. Pradhan et al. (2007) [22] modeled oxygenation of skeletal muscle as an interaction between two networks – the network of motor neurons innervating muscle fibers and the vascular network. But the model does not include glial network.

The outline of the paper is as follows. Section 2.0 describes the proposed model of neuron-astrocyte-vessel loop. Simulation results are described in Section 3.0 and discussed in the final section.

## Methods: The Model

Our model of mammalian system consists of four compartments - neuron, astrocyte, vessel and interstitium. Individual compartments were developed separately and results were matched with existing literature. To keep the presentation simple, model equations are given in the Appendix S.A; only a general description of mechanisms incorporated in each component is presented below. The integrated system consisting of 89 equations (Appendix S.A) is programmed and simulated in Matlab® 7.

### 2.1. *Neuron*

The neuron model used is a single-compartment model closely resembling the Hodgkin-Huxley model, with the primary difference being that the Nernst potentials are not constant and vary as a function of intracellular and interstitial ion concentrationsat physiological temperature of 37°C. The shortcoming of model was that it could not be used to generate firing rate below 100 Hz since it is a resonate-and-fire type neuron model [23]. This was overcome by multiplying the time constant of potassium channel (*τ_n_*) by a factor [24]. This is analogous to slowing the kinetics of potassium channel. By varying *τ_n_*, firing rates in range of 20 Hz to 80 Hz were obtained which corresponds to gamma range firing rates.

**Figure 2:**
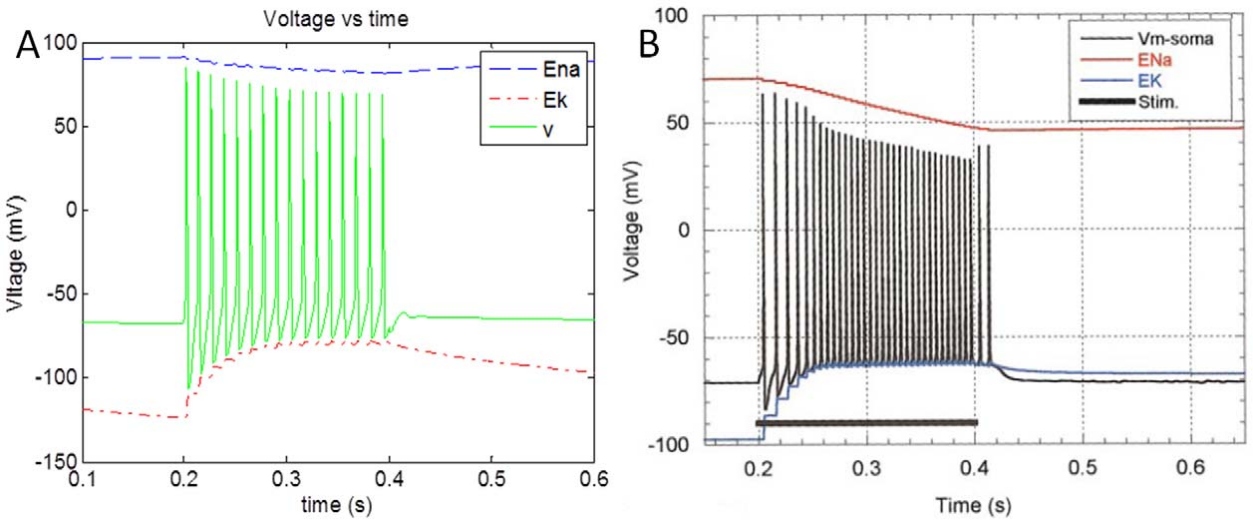
Simulation output (a) of membrane potential of neuron along with reversal potential of *Na* ^+^ (E_Na_) and *K* ^+^ (E_K_) ions when stimulated with a pulse 0.1 mA/cm^2^ current for a duration of 0.2 s (0.2 s–0.4 s). (b) Adapted from [**10**].

Variation of Nernst potentials as a function of ion concentrations, *K* ^+^ buffering, *Na* ^+^/*K* ^+^ ATPase action based on ATP – these components of the proposed neuron model were modeled after [10]. The simulated results are in accordance with the model results published in Kager et al. (2000) [10] (Fig. 2). This neuron model sustains continuous firing when injected with stimulation current above a threshold for simulation duration of 30 s.

The model of Kager et al. (2000) [10] includes voltage controlled transient and persistent sodium currents (I_Na,T_ and I_Na,P_), potassium currents (I_K,DR_ and I_K,A_), and N-methyl-D-aspartate (NMDA) receptor-controlled currents (I_NMDA_) at appropriate regions of the model cell; these currents were not included in the proposed model. The proposed minimal model of neuron is mere extension of Hodgkin-Huxley model which explains the difference in firing rates between fig. 2a (80 Hz) and fig. 2b (160 Hz).

Furthermore, the model for quantal release of glutamate described by [25] is used to model glutamate release into the synapse. Also, astrocyte recycles synaptic neurotransmitter and delivers back to neuron. Lee et al. (2009) [25] proposed a model relating action potential to glutamate release in synapse. This model was incorporated to calculate glutamate concentration in synapse.

**Figure 3:**
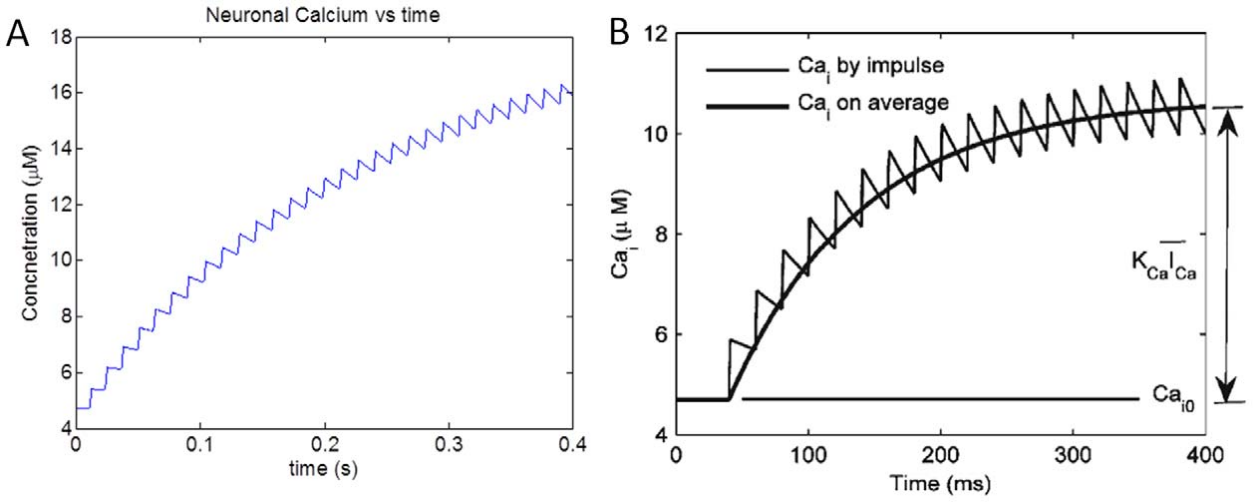
Simulation results depicting variation of (a) Neuronal *Ca^2^* ^**+**^ **concentration along with (b) as reported in** [**25**] **.**

The simulated results were found to be consistent with the results published by Lee et al. (2009) [25] (fig. 3). The minor variation in the results can be attributed to difference in firing rate of neuronal profile (∼50 Hz) from [25] and the simulated neuronal firing rate (∼80 Hz) in the proposed model. Synaptic glutamate is assumed to be cleared at a constant rate [26].

The series of events that occur in the neuron starting from current injection to neurotransmitter release are described by equations in Appendix S.A.1.

### 2.2. *Astrocyte*

Glutamate concentration in synapse is detected by astrocytes, which in turn release vasodilators. The model of [3] was adapted to describe the sequence of events from transduction of glutamate to vasodilator release from astrocyte (fig. 1). Our implementation of this component was tested and verified by simulating test conditions and matching results with corresponding results in [3].

mGluR on astrocyte detects synaptic glutamate and facilitates release of *IP_3_*inside astrocyte [27]. *IP_3_* concentration modulates *Ca^2^* ^+^ release from internal calcium stores [28]. When cytosolic *Ca^2^* ^+^ concentration crosses a threshold value, it produces *EET* which is released from astrocyte [29].

**Figure 4:**
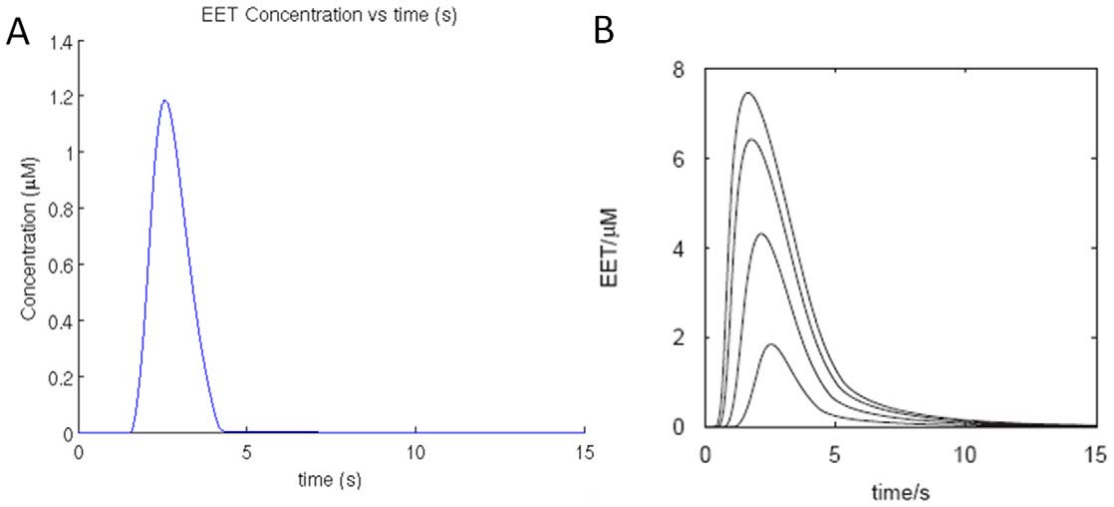
Model changes in astrocyte on application of 2 s pulses of glutamate (a) resulting change in *EET* concentration in extracellular space between astrocyte and smooth muscle cell, in the proposed model. (b) the corresponding result reported by Bennett et al., (2008) [**3**]. The lowest trace in (b) is comparable with the graph in (a).

This model was simulated with various concentrations of glutamate pulses for 2 s as performed by Bennett et al. (2008) [3] in the control experiment and the results obtained are presented below (fig. 4).

These results are matched with the simulation results from [3] (fig. 4b). The slightly lower *EET* profile in our model can be attributed to omission of diffusion kinetics. Instead a degradation term is introduced for removal of *EET* (S.A.2) which follows first order Michaelis–Menten kinetics. The parameters of this degradation term are fine-tuned to obtain approximate match with the control experiment. Furthermore, the effect of *EET* concentration on smooth muscle is controlled by passing it through a sigmoidal function.

*EET* is a potent vasodilator that opens the*K* ^+^ channels of vascular smooth muscle membrane releasing *K* ^+^ ions from the smooth muscle cell which hyperpolarizes the smooth muscle cell and leads to muscle relaxation, vessel dilation and increased glucose flux from the vessel [30]. Events that begin with binding of glutamate with *mGluR* on astrocyte, culminating in the release of *EET*, are described by equations in Appendix S.A.2.

### 2.3. *Vessel*

We assume that smooth muscle cell membrane voltage (*V_SM_*)has a linear relation with vessel radius. The maximum *V_SM_* of −30 mV (resting condition) corresponds to the minimum vessel radius of 8 µm (contracted state) and minimum V_SM_ of −75 mV (hyperpolarized state) corresponds to maximum vessel radius of 30 µm (dilated state). Vessel dilation enhances glucose flux into the astrocytes and initiates metabolic feedback. Mangia et al., (2009) [31] proposed a model for metabolic feedback to neuron and astrocytes. We adopted this model to describe metabolic feedback to neuron and astrocyte for a given input stimulus. This model is based on substrate affinity of glucose transporters (*GLUTs*) and monocarboxylate transporters (*MCT*) for glucose and lactate respectively. In the model of [31], blood vessel acts as a source of glucose and lactate with constant concentration. We put a constraint on glucose flux to incorporate activity-dependent feedback. Glucose flux is made proportional to vessel surface area which is a linear function of radius. Change in radius thus controls glucose flux from vessel. Events describing metabolic feedback, starting from glucose release from vessel, to production of *ATP* in neuron, are described by equations in Appendix S.A.3.

### 2.4. *Interstitium*

All the metabolites and extracellular ions are exchanged between neuron, astrocyte and vessel via the interstitium. This compartment in the proposed model is only involved in transporting of metabolites and exchange of ions across neuronal and astrocytic membrane through active or passive means. Another important role interstitial compartment is buffering of extracellular *K^+^* ions [10] to facilitate neuronal firing. The equations describing the metabolic exchange [31] along with equation describing the ionic exchange are described in Appendix S.A.4.

## Results

The biophysical model of neuron, astrocyte and vessel was developed as individual compartments and the behavior of individual compartments was first verified by comparing with reported results in literature, before placing in the neuron-glial-vascular loop. Simulation was run for a duration of 30 s, for a variety of inputs and initial conditions to obtain results. The model neuron displayed a variety of firing regimes like continuous firing, firing with an initial pause and burst firing (Table 1). Furthermore, a pathological condition like vessel constriction was simulated to mimic cerebrovascular condition like stroke.

**Table 1:** Neuronal firing pattern observed for various kinds of stimulation pattern.

INPUT TYPE	Input Magnitude	INITIAL atp	Behavior
**Continuous**	I_s_ = 0.1 mA/cm^2^,	[ATP]_0_ = 20 µM	Continuous Firing
**1 s Pulse**	I_s_ = 0.1 mA/cm^2^,	[ATP]_0_ = 20 µM	Return to resting state
**Continuous, subthreshold**	I_s_ = 0.05 mA/cm^2^	[ATP]_0_ = 20 µM	Firing with Initial Pause
**1 s Pulse**	I_s_ = 0.1 mA/cm^2^	[ATP]_0_ = 5 µM	Bursting
**Continuous, Vessel Oscillation**	I_s_ = 0.1 mA/cm^2^		
**2) 0.2 Hz, Maximum Radius 30 µm for 0.5 s**	[ATP]_0_ = 5 µM	Forced Vessel Oscillations	

### 3.1. Continuous Firing

**Figure 5:**
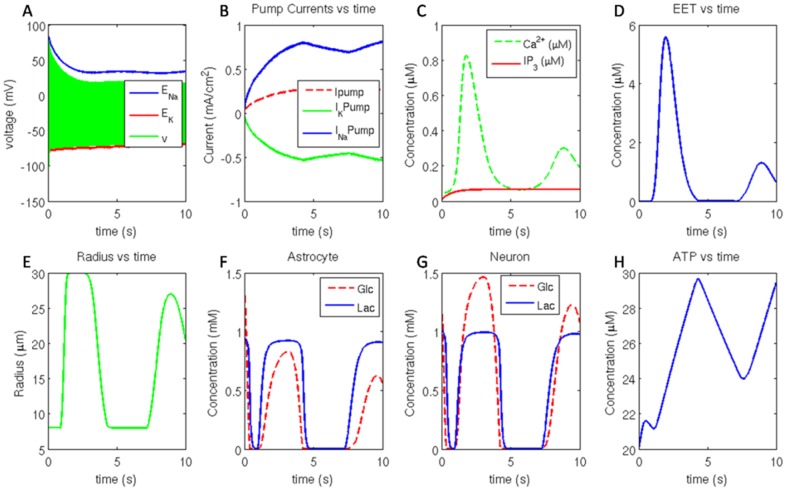
(A) Neuronal membrane potential bound by reversal potential of sodium and potassium channel, (B) *Na* ^+^/*K* ^+^ ATPase pump current, *Na* ^+^ (+ve) pump current and *K* ^+^ (−ve) pump current. (C) astrocytic*IP_3_* and *Ca^2^* ^**+**^ **concentration and the corresponding (D)** ***EET*** **released. (E) vessel radius, (F) glucose (** ***Glc*** **) and lactate (** ***Lac*** **) concentration in astrocyte. (G) glucose (** ***Glc*** **) and lactate (** ***Lac*** **) concentration in neuron along with (H) cytosolic** ***ATP*** **concentration in neuron.**

Continuous firing was obtained when the neuron was stimulated beyond a threshold stimulation current of 0.07 mA/cm^2^. Fig. 5 shows various events in the model in response to a stimulation current of 0.1 mA/cm^2^ and initial [ATP] of 20 µM. The variation of membrane potential is bound by the reversal potential of sodium and potassium channels.

Since pump activity restores the disturbance of ionic gradients caused by action potential propagation, ion channel currents and pump currents are typically in the opposite direction. *Na* ^+^ channel current is negative and *K* ^+^ channel current is positive. The magnitudes of *Na* ^+^ and *K* ^+^ pump currents (fig. 5B) always maintain the ratio of 3∶2 as the *Na* ^+^/*K* ^+^ ATPase pump extrudes 3 *Na* ^+^ ions for uptake of 2 *K* ^+^ ions.

The change in ionic concentration across the membrane was computed by combining the channel current and the pump current, and including the buffering action of astrocytes. The deficit of single *K* ^+^ ion per restorative cycle of pump was compensated by astrocytic uptake of *K* ^+^ ions. Reversal potential (fig. 5A) of an ion channel is a function of the ratio of ion concentrations across the membrane.

Synaptic glutamate activates mGluR on astrocytes which triggers release of*IP_3_* (fig. 5C) from membrane bound *PIP_2_* into the astrocyte. *IP_3_* acts on *ER* and triggers *Ca^2^* ^+^ oscillations (fig. 5C) in astrocyte. When the cytosolic *Ca^2^* ^+^ concentration in astrocyte attains a minimum threshold, it triggers release of *EET* onto the smooth muscle cells. *EET* release (fig. 5D) is observed to follow cytosolic *Ca^2^* ^+^ concentration in astrocyte.

Action of *EET* on smooth muscle causes its hyperpolarization. This causes relaxation of smooth muscle cells and consequently vessel dilation (fig. 5E). The oscillation in *EET* concentration is manifest as variation in smooth muscle membrane potential. These oscillations in smooth muscle membrane potential are thought to cause vasomotion in microvessels [16].

Vessel dilation improves flux of glucose and lactate into the interstitium through capillaries via basal endothelium. Astrocytes uptake a large amount of glucose (fig. 5F) from interstitium which is metabolized to form lactate and pyruvate. Lactate is released into the interstitium and pyruvate is metabolized to sustain cellular functions. Neuron uptakes lactate from interstitium (fig. 5G) to fuel its metabolic requirements. Lactate is oxidized via Tricarboxylic Acid (*TCA*) cycle to generate *ATP* (fig. 5H).

The variation in *ATP* concentration (fig. 5H) modulates the *Na* ^+^/*K* ^+^ ATPase pump activity which essentially restores the ionic concentration across the neuronal membrane. *ATP* build up ensures higher pump activity and hence efficient restoration of reversal potential of ion channels.

### 3.2. Firing with Initial Pause

**Figure 6:**
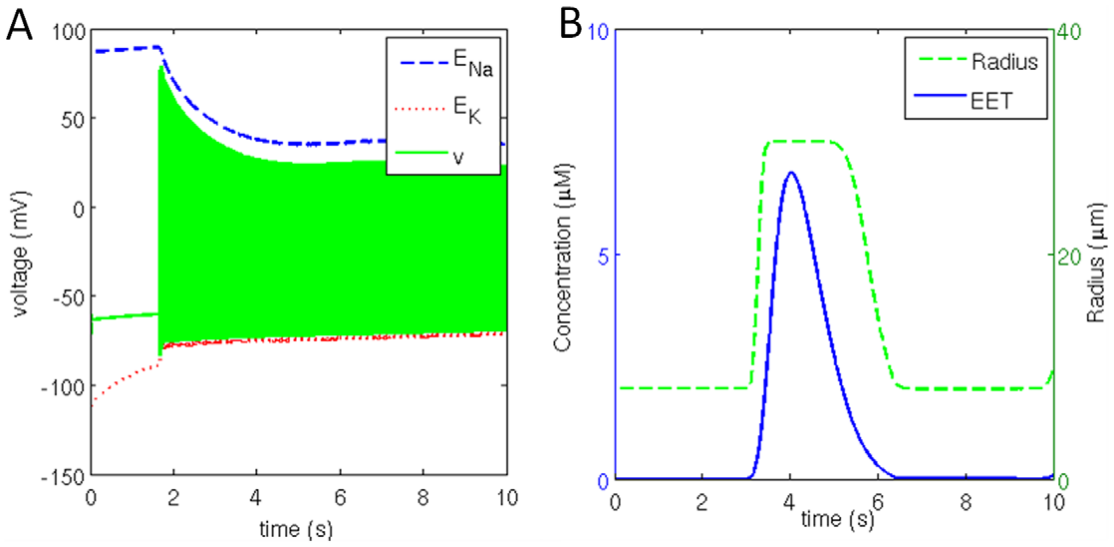
(a) Neuronal membrane potential bound by reversal potential of sodium and potassium channel along with (b) corresponding change in extracellular [EET] and the vessel radius.

Initial pause in neural firing pattern is observed when the stimulation current is below the threshold value of 0.07 mA/cm^2^ and initial [ATP] of 20 µM. For example, this firing regime is obtained when a constant stimulation current of 0.05 mA/cm^2^ is applied (fig. 6a). The neuron continues to be in resting state for a duration of 1.9 s and then fires continuously (fig. 6a). Reversal potential of *Na* ^+^ channel is stable during initial 1.9 s when the neuron is not firing but the reversal potential of *K* ^+^ is found to be increasing which can be attributed to combination of sub- threshold stimulation current and leakage current.

Glutamate releasefrom the neuron triggers release of *IP_3_* into the astrocyte. *Ca^2^* ^+^ concentration in astrocyte is maintained until the *IP_3_* acts on *ER* to release *Ca^2^* ^+^ into the astrocyte. This pulse of *Ca^2^* ^+^causes release of *EET* (fig. 6b) on to smooth muscle. Action of *EET* hyperpolarizes the smooth muscle cells and cause vessel dilation (fig. 6b). Due to vessel dilation the rates of glucose and lactate flux transfer across the endothelium into the interstitium increase. Astrocyte and neuron uptake glucose and lactate from interstitium. Neuron oxidizes lactate to produce *ATP*which fuels neuronal firing.

A steep fall in neuronal *ATP* concentration is observed after a duration of 7 s (figure not shown). The neuronal firing continues as the metabolic feedback diminishes. In this case, the *ATP* consumption is substantially more than the production rate of *ATP*. Once the *ATP* concentration becomes too low to sustain pump activity, the neuron tends to depolarize as the ionic gradients across the membrane are exhausted.

### 3.3. Pulse Stimulation

**Figure 7:**
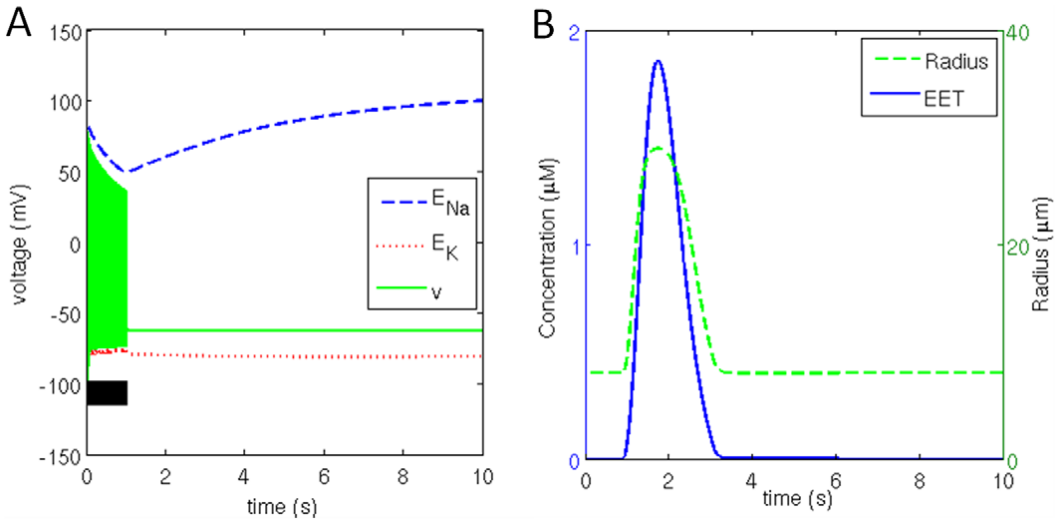
(a) Neuronal membrane potential bound by reversal potential of sodium and potassium channel for a stimulation duration of 1 s (black bar) along with (b) corresponding change in extracellular [EET] and vessel radius.

A neuron will remain in resting state in the absence of stimulation current or when injected with short pulse of current. Here we stimulate the neuron for 1 s duration with 0.1 mA/cm^2^ current which is greater than the threshold current of 0.07 mA/cm^2^, however, the initial [ATP] is 20 µM. Once the stimulus is removed the membrane potential stabilized at resting potential (fig. 7a). During the stimulation duration the neuron fired a series of action potentials and glutamate is released into the synapse.

Due to the availability of ATP, and the action of *Na* ^+^/*K* ^+^ ATPase pump, *Na* ^+^ concentration is restored within about 6 s after removal of the stimulus whereas *K* ^+^ concentration quickly attained steady states in both intracellular and extracellular compartment to maintain *E_k_* equal to −80 mV. The astrocytic buffer maintained the extracellular *K* ^+^ concentration at about 9.5 mM.

Glutamate release leads to activation of *mGluR* on astrocyte and consequent release of *IP_3_* into the astrocyte. This triggers release of a *Ca^2^* ^+^ pulse in cytosol. Due to the nonlinearity in the relationship between *Ca^2^* ^+^ and EET (Appendix S.A.2), even though *Ca^2^* ^+^ build up is roughly half in comparison to the case of continuous firing, the *EET* released (fig. 7b) is only one third in comparison to continuous firing (fig. 5D).

Although *EET* released is only third as compared to continuous firing, it is sufficient to hyperpolarize the smooth muscle cells and cause vessel dilation (fig. 7b). Vessel dilation allows glucose and lactate flux transfer into the interstitium via endothelium. Change in glucose and lactate concentration in all the compartments is observed to follow vessel dynamics.

The initial ATP reserve is exhausted to initiate firing by the end of 0.5 s which is replenished after a delay of 1 s by the metabolite feedback triggered by neuronal firing. Once the stimulus is removed, the biochemical signaling involved in vessel dilation is also arrested. No further release of metabolites is possible once the vessel constricts to a minimum radius. Once the available ATP pool is exhausted, no further generation of *ATP* is possible to maintain ionic concentration across the membrane. *ATP* concentration declines steadily, even though the neuron is not firing. Neuronal leakage current is accountable for continuous decline in reversal potential of ion channels and hence the constant consumption of *ATP*.

### 3.4. Bursting

**Figure 8:**
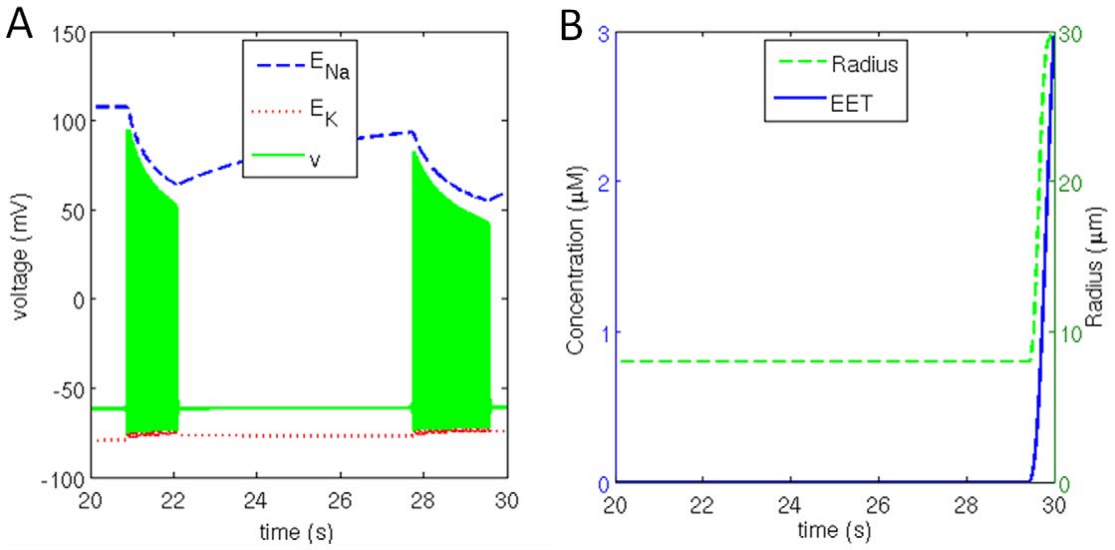
(a) Neuronal membrane potential bound by reversal potential of sodium and potassium channel along with (b) corresponding change in extracellular [EET] and subsequently the vessel radius.

Section 3.3 describes the case when the neuron is stimulated with a pulse current of magnitude 0.1 mA/cm^2^ for a duration of 1 s. In this case the initial [ATP] is reduced 5 µM from 20 µM to simulate metabolic stress. The neuron quickly returned to the resting state once the stimulus is removed and continued to remain so during the entire simulation which ran for 10 sec. However, something dramatic is observed when the simulation is continued beyond 10 sec: without any external stimulation the neuron fires a series of action potentials which resemble *bursting*. The cause of such firing is not yet established but we may speculate that neuron show bursting behavior to signal release of metabolites which can be utilized for generating *ATP*. It seems like a *SOS* call made by a neuron under metabolic stress. These action potentials (fig. 8a) have significantly large amplitudes in comparison to the continuous firing case. Consequently the synaptic glutamate concentration also seems to be slightly more than usual as the peak synaptic glutamate concentration is around 2.5 mM whereas in case of continuous firing it is only around 1.8 mM.

Interestingly the first burst does not trigger vessel dilation whereas the second burst does. It can be inferred that the synaptic glutamate must stimulate *mGluR* on astrocyte for a certain critical duration to trigger release of cytosolic *IP_3_*, sufficient enough to allow build up of cytosolic *Ca^2^* ^+^ greater than the threshold concentration of 0.1 mM, beyond which *EET* (fig. 8b) is released. Action of cytosolic *IP_3_* on *Ca^2^* ^+^ channels (on *ER*) follows slow channel activation dynamics due to which there is a delay in release of cytosolic *Ca^2^* ^+^. Action of *EET* on smooth muscle cells causes their hyperpolarization and as a consequence the vessel dilates (fig. 8b).

### 3.5. Vessel Oscillations or Vasomotion

**Figure 9:**
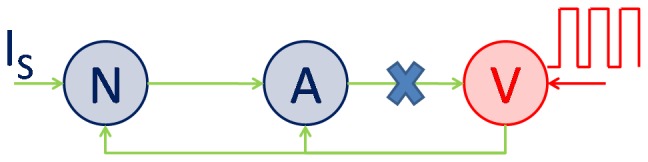
Schematic representation of induced vessel oscillations. Vessel dynamics are externally supplied and vessel activation due to astrocyte is blocked.

**Figure 10:**
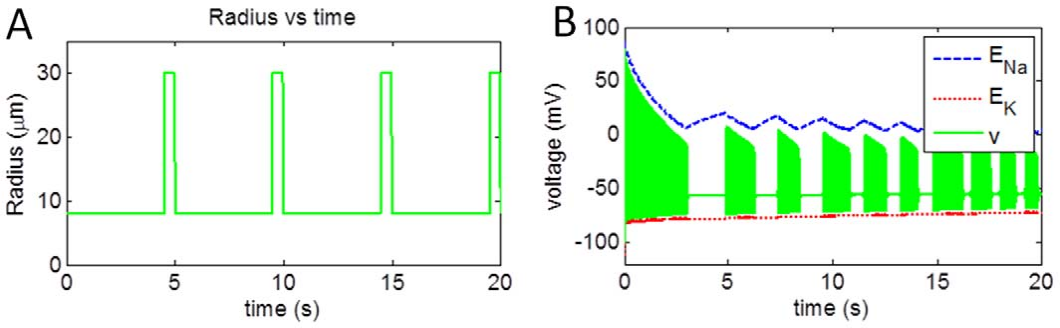
(a) Induced vessel oscillation at 0.2 Hz with vessel dilation for 0.5 s and corresponding (b) change in neuronal membrane potential bound by reversal potential of sodium and potassium channel.

The aim of this simulation is to study the effect of vascular rhythms on neural activity. Therefore, to consider the exclusive effect of the feedback from vessels, we ignored the forward influence from neuron to vessel, and only consider the feedback from the vessel to the neuron (fig. 9). The vessel was assumed to have spontaneous oscillations to representing the rhythms of *vasomotion* [16]. To investigate the effects of vascular rhythmson neuronal membrane potential, vessel oscillations were induced at 0.2 Hz with maximum vessel dilation of 30 µm for a duration of 0.5 s per cycle (fig. 10a) for a duration of 20 s. Since vessel dilation occurs only 10% of the oscillatory cycle in this simulation, it creates a metabolic stress in both neuronal and astrocytic compartments.

The neuronal firing pattern, when continuously stimulated with constant current of 0.1 mA/cm^2^ is depicted in fig. 10b. Deactivating the forward branch (neuron→vessel) while including the reverse influence (vessel→neuron) drastically influences the performance of the neuron. It can be clearly seen from the fig. 10b, that neuron fires in bursts, with approximate duration of 1 s. The neuronal firing for initial 3 s consumed all the reserve metabolites and metabolic stress is induced within neuron. Vessel dilation at 4.5 s released metabolites which were consumed by the neuron to produce ATP, initiating restoration of *Na^+^* and *K^+^* gradients. The neuronal firing at 5 s can be attributed to restoration of ionic gradients, which were soon depleted by 6 s point. The following burst of action potentials during the 7 s–8 s interval can be interpreted as an ‘SOS’ call that neuron makes in order to signal for release of metabolites while experiencing metabolic stress. This pattern continues for each cycle of vessel oscillation.

These studies open the possibility that neural firing patterns are influenced not only by stimulation from other neurons, but also by metabolic feedback from the vessels. If vascular rhythms can influence neural firing, we may begin to ask if vascular dynamics have a role in neural information processing.

## Discussion

The proposed neuron-glial-vessel model captures the entire loop of events from neuronal activity to vessel dilation to influx of energy back into the neuron to sustain its firing. To the best of our knowledge this is the first model which captures key signaling mechanisms of neuro-glial-vascular interaction in a loop. The model has compartment level segregation and offers flexibility to implement various kinds of neurons, astrocytes and vessel models. This opens the possibility to further improve the biophysical model by modifying existing pathways and by incorporating new signaling pathways.

**Figure 11:**
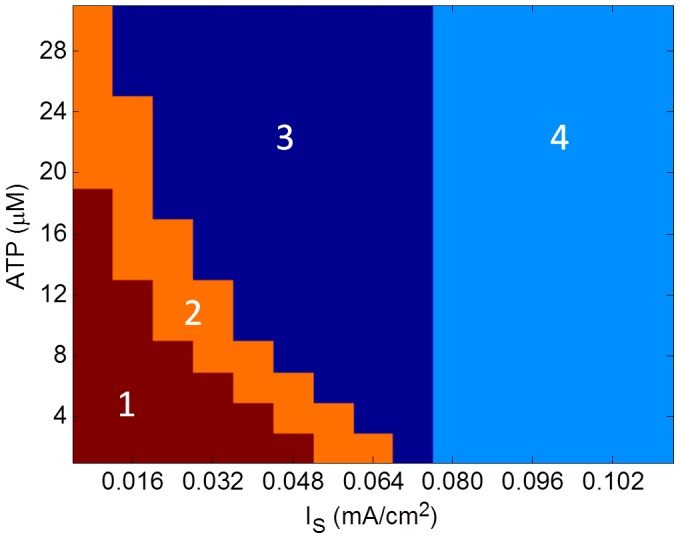
Regimes obtained for various combinations of stimulation current (I_S_) and initial [ATP]. (1) Bursting, (2)Transition phase from bursting to firing with initial pause, (3) Firing with initial pause and (4) Continuous firing.

A variety of firing patterns are obtained for different combinations of stimulation current, I_s_, and initial [*ATP*]_0_ values. In order to perform a systematic study of the firing patterns observed, we scanned the space of I_s_, and [*ATP*]_0_ and noted that the observed firing regimes can be broadly classified into 4 categories (fig. 11). According to fig. 11, for low continuous stimulation current and initial [ATP], bursting behavior is observed (fig. 11, region 1) which shifts towards firing with initial pause (fig. 11, region 3) through a transition phase (fig. 11, region 2). On increasing the stimulation current beyond 0.07 mA/cm^2^, continuous firing is observed (fig. 11, region 4).

Continuous application of a sub-threshold stimulation current to neuron resulted in firing with an initial pause. This pause is observed perhaps because the neuron acts like an ‘integrator’ in the present case. In a standard Hodgkin-Huxley model, the neuron does not show firing activity for sub-threshold current, because the Nernst potentials (*E_Na_* and *E_K_*) are constant. But in the present model, since the ionic concentrations vary, even constant application of sub-threshold currents tend to depolarize the membrane, which causes reduction in *E_Na_* (fig. 5A). Once this reduction reaches a threshold, neuronal firing is initiated. In this regime the amplitude of stimulation current and initial *ATP* concentration in neuron determine the duration of initial pause before the onset of neuronal firing. The stimulation current raises the membrane potential by disturbing the ionic gradients whereas *ATP* fuels the *Na* ^+^/*K* ^+^ ATPase pumps and reduces the membrane potential by restoring the ionic gradients. Eventually, the stimulation current dominates over *Na* ^+^/*K* ^+^ ATPase pump activity, initiating neuronal firing. High amplitude of sub-threshold stimulation current and low initial *ATP* reserve reduce the delay in onset of firing and vice versa. After the onset of neuronal firing the neuron's behavior may be considered similar to a continuously firing neuron.

When the neuron is stimulated with a stimulation current greater than threshold current of 0.07 mA/cm^2^ for a duration of 1 s, the neuron regains polarized state after firing for initial stimulation duration of 1 s. This neuronal firing for 1 s is sufficient to trigger metabolic feedback but on removal of stimulus the neuron attains resting potential and metabolic feedback diminishes. The neuronal *ATP* reserve is consumed for maintaining resting potential. Neuron with adequate initial *ATP* reserve will be able to maintain resting potential for a longer duration in comparison to neuron with low initial *ATP* reserve. For a high *ATP* reserve (for example 20 µM) the neuron maintains resting potential, whereas when the initial *ATP* reserve is low, for instance 5 µM, the neuron switches to bursting behavior signaling demand for energy.

For a sub-threshold stimulation pulse of duration 1 s, the neuron remains in polarized state until neuronal *ATP* reserve essential for maintaining resting potential is exhausted. If the initial *ATP* is adequate, the neuron will continued to remain in resting condition whereas if the initial *ATP* is low, the neuron resorted to bursting. While bursting, the neuron must fire for a duration sufficient to cause elevation of cytosolic [*Ca^2^* ^+^] beyond a threshold in astrocyte, initiating release of *EET* as signal for vasodilation and subsequent metabolic feedback. However if neuronal bursting fails to initiate metabolic feedback, the ionic gradients will be destroyed, rendering neuronal firing impossible.

In Section 3.5, effects of vessel oscillations on neuronal firing pattern are studied. Small vessels are known to produce low-frequency, spontaneous oscillations known as *vasomotion* [16]. In the model, the vessel oscillation is induced at frequency 0.2 Hz with maximum vessel radius of 30 µm for a duration of 0.5 sec during a full cycle duration of 5 sec. In this case, bursting pattern was observed. This pattern of bursting closely follows the pattern of vessel oscillations. Every dilation phase of vessel is followed immediately by a long burst, and a couple of shorter bursts of action potentials (fig. 10b).

The simulation results place emphasis on the effect of vessel dynamics on neuronal firing and suggest that computation in brain may be more comprehensively understood in terms of neuron, astrocyte and vessel interaction rather than neuronal activity alone. For sub-threshold stimulation current, the combination of initial *ATP* concentration and stimulation current determines the duration of initial pause before the onset of neuronal firing. Under metabolic stress, the neuron observes a relatively longer delay before the onset of firing for lower value of sub-threshold stimulation current. With increase in initial [*ATP*] the neuron exhibits a transition from bursting to firing with initial pause (fig. 11). Concentration of *ATP* modulates the *Na* ^+^/*K* ^+^ ATPase pump activity for restoration of ionic gradients and competes with stimulation current which degrades the ionic gradients. Initial neuronal *ATP* concentration also determines the time lag observed before onset of bursting. Greater the *ATP* reserve, longer is the delay observed before onset of the first burst. It has been observed that spiking frequency during bursting is around 40 Hz. The inter-burst duration reduces with every subsequent burst when the metabolic feedback is insufficient to maintain the ionic gradients. However, the inter-burst duration increases when metabolic feedback is sufficient to maintain resting potential of neuron.

The proposed model uses a modification of Hodgkin-Huxley model which was a model of the squid axon. It would be interesting to see if the results described here will be valid with a mammalian neuronal model. To this end we have simulated a model of hippocampal CA1 neuron from (Kager et al 2000) and inserted it in the complete neuron-astrocyte-vessel loop (Supplement S). The astrocyte and vessel components of the model are not altered. Simulations show that the new model shares many features with the model described in the previous sections. Neural firing patterns depend on the input stimulation current and initial [ATP]. In the case of firing with an initial pause, the pause duration is found to depend on initial [ATP]. Under conditions of metabolic deficiency (low [ATP]), and constricted vessel, vasomotion rhythms are seen to influence amplitude of neural action potentials. However, one key difference between the current model and the CA1-neuron-based model is that the neuron does not show bursting behavior; only continuous firing and firing with initial pause are observed. (See Supplement section S2 for more details).

This model captures the essential behavior of each compartment even though all the biophysical pathways are not considered. Neurons uptake neurotransmitters via astrocyte [1] and we have considered a probabilistic model for this phenomenon instead of a detailed model describing the kinetics of this process. In this model, diffusion of molecules has been neglected but can be implemented for a system with well-defined boundaries. Neurons, astrocytes and endothelial cells surrounding the vessels, upon activation, release nitric oxide which is a potent vasodilator [2] for upstream vasculature [32]. As the proposed system is a lumped model and does not model space, diffusion of *NO* is not incorporated. Action of vasoactive molecules released by astrocytes upon activation which are essentially arachidonic acid derivatives (except *EET*) has been neglected but can be implemented to fit experimental data. Furthermore, to simulate vessel dynamics we used a lumped model and there is no explicit representation of endothelium and smooth muscle dynamics. Metabolic feedback due to vessel dynamics does not take into account diffusive and convective transport of glucose and lactate through various compartments. Also, this model does not incorporate oxygen delivery to various tissues and assumes that sufficient oxygen is available for oxidation of glucose and lactate in neuron and astrocyte.

Furthermore, compartmental modeling allows creation of a network level model which may provide deeper insight into how brain functions. The network level model will further encourage developing models for simulating neurovascular disorders and investigating metabolic basis of neural disorders. Such network models would suggest that brain's computation as being performed, not solely by a network of neurons, but by three networks – neural, astrocytic and vascular - working in tandem. Recent work shows that astrocytes also exhibit “tuned responses” to visual stimuli, and therefore may be thought to be involved in information processing [33]. Our present modeling exercise envisages that vessels also might exhibit tuned responses. On that account, even vessels can then be thought of as performing computation and information processing, describable perhaps as *vascular computation*. These intriguing possibilities can be the subject matter of future computational and experimental studies on networks underlying cerebral circulation.

## Supporting Information

Supplement S1 **Model with hippocampal CA1 neuron in neuron-astrocyte-vessel loop.**
(DOCX)

Appendix S1 **List of equations and constants.**
(DOCX)

Figure S1 (TIF)

Figure S2 (TIF)

Figure S3 (TIF)

Figure S4 (TIF)

Figure S5 (TIF)

Figure S6 (TIF)
