# Supplementary material for: A Computational Model of Neuro-Glio-Vascular Loop Interactions
Source: PLoS One. 2012 Nov 20;7(11):e48802. doi: 10.1371/journal.pone.0048802 (PMC3502400; doi:10.1371/journal.pone.0048802)
Supplement: Supplement S1 — Model with hippocampal CA1 neuron in neuron-astrocyte-vessel loop. (DOCX) [file pone.0048802.s001.docx]

## Supplement S: A computational model of neuro-glio-vascular loop interactions

The following firing regimes are obtained by replacing the modified Hodgkin Huxley model used in the original manuscript with the model of CA1 hippocampal neuron from (Kager et al. 2000). In comparison to the model presented in the manuscript the neuron model of (Kager et al 200) comprises additional ion channels currents: Persistent *Na*^+^ current, Transient *K*^+^ current and *NMDA* mediated receptor *Na*^+^ and *K*^+^ currents. However, only the soma compartment was used in the model and no explicit representation of the dendritic compartment is implemented.

The aim of this study was to replicate the findings presented in the (original) manuscript with mammalian neuron model. Also, this further supports the robustness of the proposed neuron-glial-vascular model as the parameters representing the individual compartments can be modified to fit the system of interest. The current findings are also valid for the CA3 hippocampal pyramidal neuron model presented by Migliore et al. 1999 and Traub et al. 1991.

As in the previous model the input parameters varied are:

1. Input current

2. Initial value of [ATP] in the neuron

The firing regimes obtained with the previous model were:

1. bursting

2. firing with an initial pause

3. continuous firing

The new model does not exhibit bursting but exhibits

1. firing with an initial pause

2. continuous firing

## S.1. Continuous Firing

Continuous firing was obtained when the neuron was stimulated beyond a threshold stimulation current of 0.01 mA/cm^2^ at initial [ATP] of 50µM. Fig. S1A shows various events in the model in response to a stimulation current of 0.1 mA/cm^2^ and initial [ATP] of 50µM. The threshold stimulation current is 0.01 mA/cm^2^ in the present model, lesser than the corresponding value in the original model. This is possible since the threshold stimulation current is an intrinsic property of the neuron. The response of astrocyte and metabolic feedback is unaffected.

### S.2. Firing with Initial Pause

Initial pause in neural firing pattern is observed when the stimulation current is below the threshold value of 0.01mA/cm^2^ and initial [ATP] is 2µM. For example, this firing regime is obtained when a constant stimulation current of 0.005mA/cm^2^ is applied (fig. S2A). The neuron continues to be in resting state for a duration of 63s and then begins to fire (fig. S2A). In another simulation with initial [ATP] of 10µM the neuron begins firing after 70.8s (fig. S3A) implying increase in initial neuronal [ATP] delays the onset of neuronal firing. This sudden onset of firing can be attributed to reduction in *Na*^+^/*K*^+^ ATPase pump activity (figs. S2B and S3B) caused by reduction of [ATP] (figs. S2H and S3H) in neuron causing elevation in resting membrane potential. The elevation of resting membrane potential causes spontaneous onset of neuronal firing to initiate metabolic feedback for restoring [ATP] and *Na*^+^/*K*^+^ ATPase pump currents (figs. S2B and S3B). The consistent increase in reversal potential of *K*^+^ is observed till the onset of neuronal firing at 63s; can be attributed to combination of sub- threshold stimulation current, NMDA and leakage currents.

This increased pause duration observed with increased initial [ATP] may be explained as follows. There is a certain competition between sub-threshold stimulation current (STSC) and pump activity. STSC just by itself does not initiate firing; it is the deficiency in neuronal metabolic reserve which is causing the firing by destroying the ionic gradients and reducing firing threshold. STSC is trying to increase resting potential and therefore reduce firing threshold, whereas pump currents are trying to restore the ionic gradients and increase firing threshold. For high STSC and low initial [ATP] the pump activity is insufficient to maintain the ionic gradients and therefore causes neuronal firing. When STSC is low and initial [ATP] is high, the pump activity is high and maintains the ionic gradients for a longer time. So the ionic gradients converge after a longer delay, thereby delaying onset of neuronal firing.

### S.3. Pulse Stimulation

Here the neuron is stimulated for a duration of 1s with 0.1mA/cm^2^ current which is greater than the threshold current of 0.01mA/cm^2^, however, the initial [ATP] is 10µM. Once the stimulus is removed the membrane potential stabilized at resting potential (fig. S4A). However, in this simulation NMDA current was set to zero to avoid any residual firing beyond the stimulation duration. The total firing duration of 1.2s was observed in presence of NMDA current as depicted in fig S4B.

### S.4. Vessel Oscillations or Vasomotion

The effects of vascular rhythms on neuronal membrane potential is investigated by inducing vessel oscillations at 0.2 Hz with maximum vessel dilation of 30 µm for a duration of 0.5s per cycle (fig. S5A) and 2.5s per cycle (fig. S5C) for simulation duration of 20s as mentioned in the manuscript. The stimulation current is 0.1 mA/cm^2^. The effect of the vessel oscillation can be observed in the change in the reversal potential of *Na*^+^ and the consequent reduction of action potential amplitude in fig. S5B and fig. S5D. Initial [ATP] was set to 2μM to simulate for metabolic stress to see the effects of vessel oscillations. As an effect the neuron continues firing with amplitude modulated by vessel oscillation frequency and duration. In fig. S5D the neuron recovers from metabolic stress and unlike in fig. S5B the neuron marginally manages to continue firing with reduced amplitude. These findings echo the findings related to the effect of vasomotion on neural firing patterns presented in the original manuscript.

### S.5. Effect of initial [ATP] on neuronal excitability

To study the effect of initial [ATP] on neuron excitability the stimulation current was set to 0.007 mA/cm2 with initial [ATP] of 10μM and 50μM. Also, NMDA currents were set to zero to avoid any presynaptic current which may confound the effects of stimulation current. The observed neuronal firing patterns are displayed in fig. S6A and fig. S6B for initial [ATP] of 10μM and 50μM respectively. Neuronal firing for low initial [ATP] of 10 μM is an indication of insufficient neuronal ATP reserve to sustain *Na*^+^/*K*^+^ ATPase pump activity for maintaining neuronal resting membrane potential. However, when initial [ATP] is set to 50μM the neuron does not exhibit firing and maintains resting potential. Neuron with a low [ATP] reserve is vulnerable to firing even though when stimulated with subthreshold stimulation current. This observation suggests neuron excitability is modulated by initial neuronal [ATP].

Comment

With Kager et al. (2000) neuron model burst firing was not observed when the model is simulated in a loop. To observe bursting phenomenon further characterization of CA1 hippocampal pyramidal neuron model in the loop is required.

Equations of CA1 hippocampal pyramidal neuron model from Kager et al. 2000 introduced in the proposed model.

| **1 Persistent Sodium Current** |
| --- |
| $InaP=gnap*\left( mnap^{2} \right)*hnap*\left( v-vna \right)$ |
| 2 |
| $mnapinf=\frac{1}{1+exp\left( -\left( 0.143*v+5.67 \right) \right)}$ |
| 3 Activation rate constant for gating variable hnap at membrane potential *v* |
| $alphahnap=5.12*\left( {10}^{-8} \right)*exp\left( -\left( 0.056*v+2.94 \right) \right)$ |
| 4 Inactivation rate constant for gating variable hnap at membrane potential *v* |
| $betahnap=1.6*\frac{{10}^{-6}}{1+exp\left( -\left( 0.2*v+8 \right) \right)}$ |
| **5 Transient potasssium current** |
| $Ika=gka*\left( mka^{2} \right)*hka*\left( v-vk \right)$ |
| 6 Activation rate constant for gating variable mka at membrane potential *v* |
| $alphamka=0.02*\frac{-v-56.9}{exp\left( -\left( 0.1*v+5.69 \right) \right)-1}$ |
| 7 Inactivation rate constant for gating variable mka at membrane potential *v* |
| $betamka=0.0175*\frac{v+29.9}{exp\left( 0.1*v+2.99 \right)-1}$ |
| 8 Ativation rate constant for gating variable hka at membrane potential *v* |
| $alphahka=0.016*exp\left( -\left( 0.056*v+4.61 \right) \right)$ |
| 9 Inactivation rate constant for gating variable hka at membrane potential *v* |
| $betahka=\frac{0.5}{1+exp\left( -\left( 0.2*v+11.98 \right) \right)}$ |
| **10 Sodium current due to synaptic NMDA receptor activation.** |
| $InaNMDA=gnmda*mnmda*hnmda*\frac{v-vna}{1+0.33*Mg*exp\left( -\left( 0.07*v+0.7 \right) \right)}$ |
| **11 Potassium current due to synaptic NMDA receptor activation.** |
| $IkNMDA=gnmda*mnmda*hnmda*\frac{v-vk}{1+0.33*Mg*exp\left( -\left( 0.07*v+0.7 \right) \right)}$ |
| 12 |
| $mnmdainf=\frac{1}{1+exp\left( \frac{Kha-Ko}{Ksa} \right)}$ |
| 13 |
| $hnmdainf=\frac{1}{1+exp\left( \frac{Ko-Khi}{Ksi} \right)}$ |
| **14 Delayed rectifier *K*^+^ channel current instead of squid axon *K*^+^ channel current.** |
| $Ik= gkmax*\left( nN^{2} \right)*\left( v-vk \right)$ |
| 15 Activation rate constant for gating variable nN at membrane potential *v* |
| $alphan = 0.016*(-34.9-v)/(exp(-(0.2*v+6.98))-1)$ |
| 16 Inactivation rate constant for gating variable nN at membrane potential *v* |
| $betan = 0.25*exp(-(0.025*v+1.25)$ |
| **15 Transient *Na*^+^ channel current instead of squid axon *Na*^+^ channel current.** |
| $Ina= gnamax*m^{3}*hN *\left( v-vna \right)$ |
| 16 Activation rate constant for gating variable m at membrane potential *v* |
| $alpham = -0.32*\frac{51.9+v}{exp\left( -\left( 0.25*v+12.975 \right) \right)-1}$ |
| 17 Inactivation rate constant for gating variable m at membrane potential *v* |
| $betam = 0.28*\frac{v+24.89}{exp\left( \left( 0.2*v+4.978 \right) \right)-1}$ |
| 18 Inactivation rate constant for gating variable hN at membrane potential *v* |
| $alphah = 0.128*exp\left( -\left( 0.056*v+2.94 \right) \right)$ |
| 19 Inactivation rate constant for gating variable hN at membrane potential *v* |
| $betah =\frac{4}{1+exp\left( -\left( 0.2*v+6 \right) \right)}$ |

Figure S1: Neuron exhibiting continuous firing. Stimulation current =0.1 mA/cm^2^ and initial [ATP] = 50µM. Threshold stimulation current =0.01 mA/cm^2^. Fig. S1A shows various events in the model in response to a (A) Neuronal membrane potential bound by reversal potential of sodium and potassium channel, (B) *Na*^+^/*K*^+^ ATPase pump current, *Na*^+^ (+ve) pump current and *K*^+^ (-ve) pump current. (C) astrocytic *IP_3_* and *Ca^2^*^+^ concentration and the corresponding (D) *EET* released. (E) vessel radius, (F) glucose (*Glc*) and lactate (*Lac*) concentration in astrocyte. (G) glucose (*Glc*) and lactate (*Lac*) concentration in neuron along with (H) cytosolic *ATP* concentration in neuron.

Figure S2: Neuron exhibiting firing with initial pause. Stimulation current = 0.005mA/cm^2^, initial [ATP]= 2μM (A). Neuronal membrane potential bound by reversal potential of sodium and potassium channel, (B) *Na*^+^/*K*^+^ ATPase pump current, *Na*^+^ (+ve) pump current and *K*^+^ (-ve) pump current. (C) astrocytic *IP_3_* and *Ca^2^*^+^ concentration and the corresponding (D) *EET* released. (E) vessel radius, (F) glucose (*Glc*) and lactate (*Lac*) concentration in astrocyte. (G) glucose (*Glc*) and lactate (*Lac*) concentration in neuron along with (H) cytosolic *ATP* concentration in neuron.

Figure S3: Neuron exhibiting firing with initial pause. Stimulation current = 0.005mA/cm^2^, Initial [ATP]= 10μM. (A) Neuronal membrane potential bound by reversal potential of sodium and potassium channel, (B) *Na*^+^/*K*^+^ ATPase pump current, *Na*^+^ (+ve) pump current and *K*^+^ (-ve) pump current. (C) astrocytic *IP_3_* and *Ca^2^*^+^ concentration and the corresponding (D) *EET* released. (E) vessel radius, (F) glucose (*Glc*) and lactate (*Lac*) concentration in astrocyte. (G) glucose (*Glc*) and lactate (*Lac*) concentration in neuron along with (H) cytosolic *ATP* concentration in neuron.

Figure S4: Effect of NMDA currents on neural firing when stimulated by a pulse current. Neuronal membrane potential bound by reversal potential of sodium and potassium channel for a stimulation duration of 1s (black bar) without NMDA current (A) and with NMDA current (B).

Figure S5: Effect of vasomotion on neural firing. Induced vessel oscillation at 0.2 Hz with vessel dilation for 0.5s (A) and 2.5s (C) with corresponding change in neuronal membrane potential (B and D respectively) bound by reversal potential of sodium and potassium channel. The vasomotion rhythm can be seen to influence the amplitude of action potentials.

Figure S6: Effect of initial [ATP] on firing response. Neuronal membrane potential bound by reversal potential of sodium and potassium channel for initial [ATP] of 10μM (A) and 50μM (B) without NMDA current for stimulation current of 0.007 mA/cm^2^.

## Reference

1. Kager, H., Wadman, W. J., & Somjen, G. G. (2000). Simulated Seizures and Spreading Depression in a Neuron Model Incorporating Interstitial Space and Ion Concentrations. *Cell*, *27710*, 495-512.
2. Jolivet, R., Magistretti, P.J., & Weber, B. (2009). Deciphering Neuron-Glia Compartmentalization in Cortical Energy Metabolism. *Front Neuroenergetics*. 2009; 1:4. doi:  10.3389/neuro.14.004.2009
3. Traub, R. D., Wong R. K., Miles R., & Michelson H. (1991). A model of a CA3 hippocampal pyramidal neuron incorporating voltage-clamp data on intrinsic conductances. AJP - JN Physiol *August 1, 1991 vol. 66 no. 2 635-650*
