# Supplementary material for: A Computational Model of Neuro-Glio-Vascular Loop Interactions
Source: PLoS One. 2012 Nov 20;7(11):e48802. doi: 10.1371/journal.pone.0048802 (PMC3502400; doi:10.1371/journal.pone.0048802)
Supplement: Appendix S1 — List of equations and constants. (DOCX) [file pone.0048802.s002.docx]

# APPENDIX S.A

# List of Equations

| S.A.1. Neuron |
| --- |
| 1.01 Reversal Potential of *K^+^* channel (Hodgkin & Huxley, 1952) |
| $v_{k} = 26.70*log\left( \frac{[K_{o}]}{{[K}_{i}N]} \right)$ |
| 1.02 Reversal Potential of *Na^+^* channel (Hodgkin & Huxley, 1952) |
| $v_{Na}= 26.70*log\left( \frac{{[Na}_{o}]}{{[Na}_{i}]} \right)$ |
| 1.03 Conductance of *K^+^* channels (Hodgkin & Huxley, 1952) |
| $g_{k} = g_{KMax}*n_{N}^{4}$ |
| 1.04 Conductance of *Na*^+^ channels (Hodgkin & Huxley, 1952) |
| $g_{Na}= g_{NaMax}*m^{3}*hN$ |
| 1.05 Activity of Na+/K+ ATPase pump (Modified, Kager et al., 2000 ) |
| $A_{pump}= \left( \left( 1+\frac{K_{mK}}{{[K}_{o}]} \right)^{-2} \right)*\left( \left( 1+\frac{K_{mNa}}{[Na_{i}]} \right)^{-3} \right)*\left( \left( 1+\frac{K_{ATP}}{[ATP]} \right)^{-1} \right)$ |
| 1.06 *K^+^* current of *Na^+^*/*K^+^* ATPase pump (Kager et al., 2000) |
| $I_{kp}=-2*I_{max}*A_{pump}$ |
| 1.07 *K^+^* current of *Na^+^*/*K^+^* ATPase pump (Kager et al., 2000) |
|  |
| $I_{Nap}=3*I_{max}*A_{pump}$ |
| 1.08 *K^+^* channel current (Hodgkin & Huxley, 1952) |
| $I_{K}=g_{K}*\left( v-v_{K} \right)$ |
| 1.09 *Na^+^* channel current (Hodgkin & Huxley, 1952) |
| $I_{Na}=g_{Na}*\left( v-v_{Na} \right)$ |
| 1.10 Total *K^+^* current (Kager et al., 2000) |
| $I_{KTot}=I_{K}+I_{Kp}$ |
| 1.11 Total *Na*^+^ current (Kager et al., 2000) |
| $I_{NaTot}=I_{Na}+I_{Nap}$ |
| 1.12 Change in [*K^+^*] in neuron (Kager et al., 2000) |
| $\frac{d{[K}_{i}N]}{dt}=-\left( I_{KTot}*\frac{s}{F*V_{i}} \right)*1000$ |
| 1.13 Change in [*Na^+^*] in neuron (Kager et al., 2000) |
| $\frac{d[Na_{i}]}{dt}=-\left( I_{NaTot}*\frac{s}{F*V_{i}} \right)*1000$ |
| 1.14 Total ionic conductance of neuron (Hodgkin & Huxley, 1952) |
| $g_{Tot}=g_{Na}+g_{K}+g_{l}$ |
| 1.15 Steady state neuronal membrane potential |
| $v_{inf}=\frac{\left( g_{Na}*v_{Na}+g_{K}*v_{K}+g_{l}*v_{l} \right)+ iapp}{g_{Tot}}$ |
| 1.16 Time constant for neuronal membrane potential (Hodgkin & Huxley 1952) |
| $\tau_{v}=\frac{c_{m}}{g_{Tot}}$ |
| 1.17 Update rule for membrane potential (Hodgkin & Huxley 1952) |
| ${v(t+\Delta t)=v}_{inf}+(v(t)-v_{inf})*exp\left( -\frac{\Delta t}{\tau_{v}} \right)$ |
| 1.18 Activation rate constant for gating variable *m* at membrane potential *v* (Hodgkin & Huxley 1952) |
| $\alpha_{m}= 0.1*\frac{v+35}{1-exp\left( -\frac{v+35}{10} \right)}$ |
| 1.19 Inactivation rate constant for gating variable *m* at membrane potential *v* (Hodgkin & Huxley 1952) |
| $\beta_{m}= 4*exp\left( -0.0556*\left( v+65 \right) \right)$ |
| 1.20 Activation rate constant for gating variable *n* at membrane potential *v* (Hodgkin & Huxley 1952) |
| $\alpha_{nN}= 0.01*\frac{v+50}{1-exp\left( -\frac{v+50}{10} \right)}$ |
| 1.21 Inactivation rate constant for gating variable *n* at membrane potential *v* (Hodgkin & Huxley 1952) |
| $\beta_{nN}= 0.125*exp\left( -\frac{v+60}{80} \right)$ |
| 1.22 Activation rate constant for gating variable *h* at membrane potential *v* (Hodgkin & Huxley 1952) |
| $\alpha_{hN}= 0.07*exp\left( -0.05*\left( v+60 \right) \right)$ |
| 1.23 Activation rate constant for gating variable *h* at membrane potential *v* (Hodgkin & Huxley 1952) |
| $\beta_{hN}=\frac{1}{1+exp\left( -0.1*\left( v+30 \right) \right)}$ |
| 1.24 Time constant for gating variable *m* (Hodgkin & Huxley 1952) |
| $\tau_{m}=\frac{1}{\alpha_{m}+\beta_{m}}$ |
| 1.25 Time constant for gating variable *h* (Hodgkin & Huxley 1952) |
| $\tau_{hN}=\frac{1}{\alpha_{h}+\beta_{h}}$ |
| 1.26 Time constant for gating variable *n* (Hodgkin & Huxley 1952) |
| $\tau_{nN}=\frac{1}{\alpha_{n}+\beta_{n}}$ |
| 1.27 Steady state value of gating variable *m* (Hodgkin & Huxley 1952) |
| $m_{inf} =\frac{\alpha{}_{m}}{\alpha_{m}+\beta_{m}}$ |
| 1.28 Steady state value of gating variable *h* (Hodgkin & Huxley 1952) |
| ${hN}_{\inf}=\frac{\alpha_{h}}{\alpha_{m}+\beta_{m}}$ |
| 1.29 Steady state value of gating variable *n* (Hodgkin & Huxley 1952) |
| ${nN}_{inf}=\frac{\alpha_{n}}{\alpha_{n}+\beta_{n}}$ |
| 1.30 Update rule for gating variable *m* (Hodgkin & Huxley 1952) |
| $m(t+\Delta t)=m_{inf}+\left( m(t)-m_{inf} \right)*exp\left( -\frac{\Delta t}{\tau_{m}} \right)$ |
| 1.31 Update rule for gating variable *h* (Hodgkin & Huxley 1952) |
| $hN(t+\Delta t)=h_{inf}+\left( hN(t)-h_{inf} \right)*exp\left( -\frac{\Delta t}{\tau_{h}} \right)$ |
| 1.32 Update rule for gating variable *n* (Hodgkin & Huxley 1952) |
| $nN(t+\Delta t)=n_{inf}+\left( nN(t)-n_{inf} \right)*exp\left( -\frac{\Delta t}{\tau_{n}} \right)$ |
| 1.33 Change of [*Ca*^2+^] in neuron (Lee et al., 2009) |
| $\frac{d[Ca_{N}]}{dt}=\frac{\left( \left( -[Ca_{N}]+[Ca_{No}] \right)+K_{CaN}*I_{Ca} \right)}{\tau_{Ca}}$ |
| 1.34 Release probability of vesicles (Lee et al., 2009) |
| $P_{rel}=P_{rel,max}*\frac{{{[Ca}_{N}]}^{4}}{{{[Ca}_{N}]}^{4}+ K_{rel,1/2}^{4}}$ |
| 1.35 Recovery rate from empty to releasable state (Lee et al., 2009) |
| $K_{rec}=K_{rec0}+\left( K_{rec0,max}-K_{rec0} \right)*\frac{[Ca_{N}]}{[Ca_{N}]+K_{rec,1/2}}$ |
| 1.36 Ratio of releasable vesicles (Lee et al., 2009) |
| $\frac{dR_{rel}}{dt}=\left( K_{rec}*\left( 1-R_{rel} \right)-P_{rel}*I_{Ca}*R_{rel} \right)$ |
| 1.37 Synaptic glutamate concentration (Modified fron Lee et al., 2009) |
| $\frac{d\left[ Glu\left( t \right) \right]}{dt}=\frac{n*N_{tot}*R_{rel}*P_{rel}*I_{Ca}}{N_{A}} -\frac{\left[ Glu \right]}{\tau_{G}}$ |
| 1.38 Neuron V_max_ hexokinase (Mangia et al.,2009) |
| $nVmh = GlycolyticRatio*\frac{\frac{nn}{2.95}}{nRoi}$ |
| 1.39 Neuron V_max_ lactate consumption (Mangia et al.,2009) |
| $nVml = 1.22*\frac{OxidativeRatio}{nRLoi}$ |
| 1.40 Neuronal lactate oxidation (Mangia et al.,2009) |
| $N_{LOxi}=1*nVml*\frac{\frac{NL}{nV}}{Kml+\frac{NL}{nV}}$ |
| 1.41 Neuronal glucose oxidation (Mangia et al.,2009) |
| $N_{GO}=\frac{NG}{nV}*nVmh*\frac{\frac{1}{12}}{Kmh+\frac{NG}{nV}}$ |
| 1.42 Neuronal glucose utilization (Mangia et al.,2009) |
| $N_{GUt}=\frac{NG}{nV}*\frac{nVmh}{Kmh+\frac{NG}{nV}}$ |
| 1.43 Conversion of neuronal glucose to lactate (Mangia et al.,2009) |
| $N_{G2L}= 2*N_{GUt}$ |
| 1.44 Change in neuronal glucose concentration (Mangia et al.,2009) |
| $\frac{dNG}{dt}= +G_{I2N}-N_{O}-N_{GUt}$ |
| 1.45 Change in neuronal lactate concentration (Mangia et al.,2009) |
| $\frac{dNL}{dt}= +L_{I2N}-N_{LOxi}+N_{G2L}$ |
| 1.46 Rate of ATP production |
| $V_{ATP}=\left( N_{LOxi} \right)*\left( {10}^{15} \right)*.53*17*180*\frac{{10}^{-9}}{60}$ |
| 1.47 Change in neuronal ATP concentration |
| $\frac{d[ATP]}{dt}=\left( 1*\frac{V_{ATP}}{V_{i}*\left( {10}^{-15} \right)}-\left( I_{Nap}+I_{Kp} \right)*\frac{s}{F*V_{i}}*\left( {10}^{9} \right) \right)*\left( {10}^{-6} \right)$ |
| S.A.2. Astrocyte |
| 1.48 Ratio of bound to total glutamate receptors in synapse (Bennett et al., 2008) |
| $\rho=\frac{[Glu]}{K_{glut}+[Glu]}$ |
| 1.49 Ratio of activated to total G-protein receptors (Bennett et al., 2008) |
| $G =\frac{\rho+\delta}{K_{g}+\rho+\delta}$ |
| 1.50 Change in *IP_3_* concentration in astrocyte (Modified from Bennett et al., 2008) |
| $\frac{d[IP_{3}]}{dt}=rh*G-K_{deg}*[IP_{3}]$ |
| 1.51 Rate of *Ca*^2+^ concentration change due to pump uptake into ER (Bennett et al., 2008) |
| $J_{pump}=V_{max}*\left( \frac{\left[ Ca_{A} \right]^{2}}{\left[ Ca_{A} \right]^{2}+K_{p}^{2}} \right)$ |
| 1.52 Gating variable of *Ca*^2+^ activated *IP*_3_ receptors (Bennett et al., 2008) |
| $\frac{dhA}{dt}=k_{on}*\left( K_{inh}-\left( [Ca_{A}]+K_{inh} \right)*hA \right)$ |
| 1.53 Rate of *Ca*^2+^ concentration change due to release through *IP*_3_ channels (Bennett et al., 2008) |
| $J_{IP_{3}}=J_{max}*\left( \left( \frac{IP_{3}}{IP_{3}+K_{i}A} \right)*\left( \frac{[Ca_{A}]}{[Ca_{A}]+K_{act}} \right)*hA \right)^{3}*\left( 1-\left( \frac{[Ca_{A}]}{[Ca_{A}]+Ca_{ERA}} \right) \right)$ |
| 1.54 Rate of *Ca*^2+^ concentration change due to leakage from ER (Bennett et al., 2008) |
| $J_{leak}=Pl*\left( 1-\left( \frac{[Ca_{A}]}{Ca_{ERA}} \right) \right)$ |
| 1.55 Cytosolic *Ca*^2+^ concentration in astrocyte (Bennett et al., 2008) |
| $\frac{dCa_{A}}{dt}=\beta_{cyt}*\left( J_{IP_{3}}-J_{pump}+J_{leak} \right)$ |
| 1.56 Rate of production of EET (Modified from Bennett et al., 2008) |
| $\frac{d[EET]}{dt}= V_{EET}*ramp\left( [Ca_{A}]-Ca_{MinA} \right)- .2*\frac{[EET]}{[EET]+18}$ |
| 1.57 Astrocytic V_max_ lactate consumption (Mangia et al.,2009) |
| $aVml = 0.24*\frac{OxidativeRatio}{aRLoi}$ |
| 1.58 Astrocytic V_max_ hexokinase (Mangia et al.,2009) |
| $aVmh = GlycolyticRatio*\frac{n}{aRoi}$ |
| 1.59 Astrocytic lactate oxidation (Mangia et al., 2009) |
| $A_{LOxi}= 1*aVml*\frac{\frac{AL}{aV}}{Kml+\frac{AL}{aV}}$ |
| 1.60 Astrocytic glucose oxidation (Mangia et al., 2009) |
| $A_{GO}=aVmh*\frac{AG}{aV}*\frac{\frac{1}{12}}{Kmh+\frac{AG}{aV}}$ |
| 1.60 Astrocytic glucose oxidation (Mangia et al., 2009) |
| 1.61 Astrocytic glucose utilization (Mangia et al., 2009) |
| $A_{GUt}=aVmh*\frac{\frac{AG}{aV}}{Kmh+\frac{AG}{aV}}$ |
| 1.62 Conversion of astrocytic glucose to lactate (Mangia et al.,2009) |
| $A_{G2L}= 2*A_{GUt}$ |
| 1.63 Change in astrocytic glucose concentration (Mangia et al.,2009) |
| $\frac{dAG}{dt}=+G_{B2A}-G_{A2I}-A_{O}-A_{GUt}$ |
| 1.64 Change in astrocytic lactate concentration (Mangia et al.,2009) |
| $\frac{dAL}{dt}=+L_{B2A}-L_{A2I}-A_{LOxi}+A_{G2L}$ |
| S.A.3. Vessel |
| 1.65 Smooth muscle membrane potential |
| $V_{m}=5-80\frac{1}{1+e^{-2\left[ \mathrm{EET} \right]}}$ |
| 1.66 Change in vessel radius |
| $r=r_{min}+\left( r_{max}-r_{min} \right)\left[ \frac{V_{max}-V_{m}}{V_{max}-V_{min}} \right]$ |
| 1.67 Glucose flux from blood |
| $BGF=\left( \frac{r-r_{min}}{r_{max}-r_{min}} \right){[Glc]}_{B}$ |
| 1.68 Lactate flux from blood |
| $BLF=\left( \frac{r-r_{min}}{r_{max}-r_{min}} \right){[Lac]}_{B}$ |
| 1.69 Glucose flux from blood to endothelium (Mangia et al., 2009) |
| $\left[ Glc \right]_{B}=\frac{SG*\left( K+\frac{EG}{eV} \right)-\frac{EG}{eV}*\left( K+SG \right)}{K^{2}*eRoo+eRoi*K*SG+eRio*K*\frac{EG}{eV}+eRee*SG*\frac{EG}{eV}}$ |
| 1.70 Glucose flux from endothelium to basal lamina (Mangia et al., 2009) |
| $G_{E2B}=\frac{\frac{EG}{eV}*\left( K+\frac{BG}{bV} \right)-\frac{BG}{bV}*\left( K+\frac{EG}{eV} \right)}{K^{2}*\frac{eRoo}{fe}+\frac{eRoi}{fe}*K*\frac{BG}{bV}+\frac{eRio}{fe}*K*\frac{EG}{eV}+\frac{eRee}{fe}*\frac{BG}{bV}*\frac{EG}{eV}}$ |
| 1.71 Lactate flux from blood to endothelium (Mangia et al., 2009) |
| $\left[ Lac \right]_{B}=\frac{SL*\left( eKL+\frac{EL}{eV} \right)-\frac{EL}{eV}*\left( eKL+SL \right)}{eKL^{2}*eRLoo+eRLoi*eKL*SL+eRLio*eKL*\frac{EL}{eV}+eRLee*SL*\frac{EL}{eV}}$ |
| 1.72 Lactate flux from endothelium to basal lamina (Mangia et al., 2009) |
| $L_{E2B}=\frac{\frac{EL}{eV}*\left( eKL+\frac{BL}{bV} \right)-\frac{BL}{bV}*\left( eKL+\frac{EL}{eV} \right)}{eKL^{2}*\frac{eRLoo}{fe}+\frac{eRLoi}{fe}*eKL*\frac{BL}{bV}+\frac{eRLio}{fe}*eKL*\frac{EL}{eV}+\frac{eRLee}{fe}*\frac{BL}{bV}*\frac{EL}{eV}}$ |
| 1.73 Glucose flux through endothelium (Mangia et al., 2009) |
| $\frac{dEG}{dt}=+BGF-G_{E2B}$ |
| 1.74 Glucose flux through basal lamina (Mangia et al., 2009) |
| $\frac{dBG}{dt}=+G_{E2B}-G_{B2A}-G_{B2I}$ |
| 1.75 Lactate flux through endothelium (Mangia et al., 2009) |
| $\frac{dEL}{dt}=-L_{E2B}+BLF$ |
| 1.76 Lactate flux through basal lamina (Mangia et al., 2009) |
| $\frac{dBL}{dt}=+L_{E2B}-L_{B2A}-L_{B2I}$ |
| S.A.4. Interstitium |
| 1.77 Buffering of interstitial *K*^+^ |
| $\frac{d{K^{+}}_{up}}{dt}=k_{1}\left[ Kbuffer \right]-k_{2}\left[ Buffer \right]K_{o}$ |
| 1.78 Change in interstitial *K*^+^ concentration (Kager et al., 2000) |
| $\frac{dK_{o}}{dt}=\left( I_{KTot}*\frac{s}{F*V_{e}} \right)*1000+\frac{d{K^{+}}_{up}}{dt}$ |
| 1.79 Change in interstitial *Na*^+^ concentration (Kager et al., 2000) |
| $\frac{dNa_{o}}{dt}=\left( I_{NaTot}*\frac{s}{F*V_{e}} \right)*1000$ |
| 1.80 Glucose flux through interstitial space (Mangia et al., 2009) |
| $\frac{dIG}{dt}=+G_{A2I}-G_{I2N}+G_{B2I}$ |
| 1.81 Lactate flux through interstitial space (Mangia et al., 2009) |
| $\frac{dIL}{dt}=-L_{I2N}+L_{A2I}+L_{B2I}$ |
| 1.82 Glucose flux from basal lamina to astrocyte (Mangia et al., 2009) |
| $G_{B2A}=\frac{\frac{BG}{bV}*\left( K+\frac{AG}{aV} \right)-\frac{AG}{aV}*\left( K+\frac{BG}{bV} \right)}{12.5*\left( K^{2}*aRoo+aRoi*K*\frac{BG}{bV}+aRio*K*\frac{AG}{aV}+aRee*\frac{BG}{bV}*\frac{AG}{aV} \right)}$ |
| 1.83 Glucose flux from astrocyte to interstitial space (Mangia et al., 2009) |
| $G_{A2I}=\frac{\frac{AG}{aV}*\left( K+\frac{IG}{iV} \right)-\frac{IG}{iV}*\left( K+\frac{AG}{aV} \right)}{{1*(K}^{2}*aRoo+aRoi*K*\frac{IG}{iV}+aRio*K*\frac{AG}{aV}+aRee*\frac{IG}{iV}*\frac{AG}{aV})}$ |
| 1.84 Glucose flux from interstitial space to neuron (Mangia et al., 2009) |
| $G_{I2N}=\frac{\frac{IG}{iV}*\left( nK+\frac{NG}{nV} \right)-\frac{NG}{nV}*\left( nK+\frac{IG}{iV} \right)}{nK*nK*nRoo+nRoi*nK*\frac{IG}{iV}+nRio*nK*\frac{NG}{nV}+nRee*\frac{IG}{iV}*\frac{NG}{nV}}$ |
| 1.85 Glucose flux from basal lamina to interstitial space (Mangia et al., 2009) |
| $G_{B2I}=\left( \frac{BG}{bV*1e15}*k_{diff} \right)-\left( \frac{IG}{iV*1e15}*k_{diff} \right)$ |
| 1.86 Lactate flux from basal lamina to astrocyte (Mangia et al., 2009) |
| $L_{B2A}=\frac{\frac{BL}{bV}*\left( aKL+\frac{AL}{aV} \right)-\frac{AL}{aV}*\left( aKL+\frac{BL}{bV} \right)}{12.5*\left( aKL^{2}*aRLoo+aRLoi*aKL*\frac{BL}{bV}+aRLio*aKL*\frac{AL}{aV}+aRLee*\frac{BL}{bV}*\frac{AL}{aV} \right)}$ |
| 1.87 Lactate flux from astrocyte to interstitial space (Mangia et al., 2009) |
| $L_{A2I}=\frac{1*\frac{AL}{aV}*\left( aKL+\frac{IL}{iV} \right)-\frac{IL}{iV}*\left( aKL+\frac{AL}{aV} \right)}{aKL^{2}*aRLoo+aRLoi*aKL*\frac{IL}{iV}+aRLio*aKL*\frac{AL}{aV}+aRLee*\frac{IL}{iV}*\frac{AL}{aV}}$ |
| 1.88 Lactate flux from interstitial space to neuron (Mangia et al., 2009) |
| $L_{I2N}=\frac{\frac{IL}{iV}*\left( nKL+\frac{NL}{nV} \right)-\frac{NL}{nV}*\left( nKL+\frac{IL}{iV} \right)}{nKL*nKL*nRLoo+nRLoi*nKL*\frac{IL}{iV}+nRLio*nKL*\frac{NL}{nV}+nRLee*\frac{IL}{iV}*\frac{NL}{nV}}$ |
| 1.89 Lactate flux from basal lamina to interstitial space (Mangia et al., 2009) |
| $L_{B2I}=\left( \frac{BL}{bV*1e15}*k_{diff} \right)-\left( \frac{IL}{iV*1e15}*k_{diff} \right)$ |

# APPENDIX S.B

# List of Constants

| $g_{KMax}=0.36\frac{mmho}{mm^{2}}$ | Peak Potassium Conductance | Hodgkin & Huxley, 1952 |
| --- | --- | --- |
| $g_{NaMax}=1.20\frac{mmho}{mm^{2}}$ | Peak Sodium Conductance | Hodgkin & Huxley, 1952 |
| $g_{l}=0.003\frac{mmho}{mm^{2}}$ | Peak Leakage Conductance | Hodgkin & Huxley, 1952 |
| $v_{l}=-54.387 mV$ | Leakage Membrane Potential | Hodgkin & Huxley, 1952 |
| $c_{m}=0.01 \frac{\mu F}{mm^{2}}$ | Membrane Capacitance | Hodgkin & Huxley, 1952 |
| $K_{mK}=3.5 mM$ |  | Kager et al. 2000 |
| $K_{mNa}=10 mM$ |  | Kager et al. 2000 |
| $Imax=0.013*100\frac{mA}{cm^{2}}$ | Maximum Na/K ATPase Pump Current | Kager et al. 2000 |
| $K_{CaN}=0.120\frac{mM}{ms}$ | Intracellular Calcium Concentration Gain per Action Potential | Lee et al. 2009 |
| $K_{ATP}=170 mM$ |  | Fitted to Kager et al. 2000 |
| $\tau_{Ca}=250 ms$ |  | Fitted to Lee et al. 2009 |
| $F=96500\frac{C}{mol}$ | Faraday’s Constant |  |
| $K_{rel,\frac{1}{2}}=0.009 mM$ | Calcium Sensitivity Regarding Transmitter Release | Lee et al. 2009 |
| $P_{rel,Max}=0.9$ | Maximum Probability of Release | Lee et al. 2009 |
| $K_{rec0}=2.2*{10}^{-2} ms^{-1}$ | Initial Recovery Rate of Synaptic Vesicle from Empty to Releasable State | Lee et al. 2009 |
| $K_{rec,Max}=2.2*{10}^{-2} ms^{-1}$ | Maximum Recovery Rate of Synaptic Vesicle from Empty to Releasable State | Lee et al. 2009 |
| $K_{rec,\frac{1}{2}}=20*{10}^{-3} mM$ | Calcium Sensitivity Regarding Transmitter Release | Lee et al. 2009 |
| $s=1586 \mu m^{2}$ | Total Surface Area of Neuron | Kager et al. 2000 |
| $V_{i}=2160 \mu m^{3}$ | Total Internal Volume of Neuron | Kager et al. 2000 |
| $OxidativeRatio =.184$ |  | Mangia et al. 2009 |
| $GlycolyticRatio =.0845$ |  | Mangia et al. 2009 |
| $K_{Glu}=1 mM$ | Dissociation Constant | Bennett et al. 2008 |
| $K_{g}=8.82$ | G-Protein Dissociation Constant | Bennett et al. 2008 |
| $K_{deg}=\frac{1.25}{1000} s^{-1}$ | IP_3_ Degradation Rate | Bennett et al. 2008 |
| $Ca_{ERA}=400\mu M$ | Ca^2+^ concentration in ER | Bennett et al. 2008 |
| $V_{max}=\frac{20}{1000}\frac{\mu M}{ms}$ | Maximum Pumping Rate of Ca^2+^ into ER | Bennett et al. 2008 |
| $K_{p}=0.24\mu M$ | Pump Dissociation Constant | Bennett et al. 2008 |
| $J_{max}=\frac{2880}{1000}\frac{\mu M}{ms}$ | Maximum Ca^2+^ Channel Current | Bennett et al. 2008 |
| $K_{i}A = 0.03\mu M$ | IP_3_ Channel Kinetic Parameter | Bennett et al. 2008 |
| $K_{act} = 0.17\mu M$ | IP_3_ Channel Kinetic Parameter | Bennett et al. 2008 |
| $k_{on}=\frac{2}{1000}\frac{\mu M}{ms}$ | IP_3_ Channel Kinetic Parameter | Bennett et al. 2008 |
| $Ca_{MinA}=0.1\mu M$ | Minimum Ca^2+^ Concentration for EET Production | Bennett et al. 2008 |
| $\beta_{cyt}=0.0244$ | Endogenous Buffer Parameter | Bennett et al. 2008 |
| $K_{inh}=0.1\mu M$ | IP_3_ Channel Kinetic Parameter | Bennett et al. 2008 |
| $P_{l}=\frac{0.0804}{1000}\frac{\mu M}{ms}$ | Determined by Steady State Balance Condition | Bennett et al. 2008 |
| $Kml = 2$ |  | Mangia et al. 2009 |
| $Kmh = 0.045$ |  | Mangia et al. 2009 |
| $V_{eet}=3*{10}^{-13}*\left( 6.022*\left( {10}^{23} \right)*\left( {10}^{-6} \right)*6*25*25*{10}^{-12} \right)*100 \frac{\mu Mol}{ms}$ | EET Production Rate | Bennett et al. 2008 |
| $aRoi=\frac{5e14}{.4}\frac{s}{m mol}$ |  | Mangia et al. 2009 |
| $n=1$ (#) |  | Mangia et al. 2009 |
| $aRLoi=6.6e13\frac{s}{m mol}$ |  | Mangia et al. 2009 |
| $aVml=0.24*\frac{OxidativeRatio}{aRLoi}$ |  | Mangia et al. 2009 |
| $aVmh=GlycolyticRatio*\frac{n}{aRoi}$ |  | Mangia et al. 2009 |
| $SG=5.5$mM | Serum Glucose Concentration | Mangia et al. 2009 |
| $r_{min}=8 \mu m$ | Minimum Vessel Radius |  |
| $r_{max}=30\mu m$ | Maximum Vessel Radius |  |
| $eKL=8$ |  | Mangia et al. 2009 |
| $SL=1mM$ | Serum Lactate Concentration | Mangia et al. 2009 |
| $fe=1$ (#) |  | Mangia et al. 2009 |
| $eRoi=\frac{13e13}{.4}\frac{s}{m mol}$ |  | Mangia et al. 2009 |
| $eRio=\frac{13e13}{.4}\frac{s}{m mol}$ |  | Mangia et al. 2009 |
| $eRee =\frac{13e13}{.4}\frac{s}{m mol}$ |  | Mangia et al. 2009 |
| $eRoo=eRoi+eRio-eRee$ |  | Mangia et al. 2009 |
| $eRLoi=2.8e15\frac{s}{m mol}$ |  | Mangia et al. 2009 |
| $eRLio = 2e15\frac{s}{m mol}$ |  | Mangia et al. 2009 |
| $eRLee=2e15\frac{s}{m mol}$ |  | Mangia et al. 2009 |
| $eRLoo=2.8e15\frac{s}{m mol}$ |  | Mangia et al. 2009 |
| $V_{e}=0.15*2160$ µL | Extracellular Volume | Kager et al. 2000 |
| $k_{1}=.0008$ |  | Kager et al. 2000 |
| $k_{2}=.\frac{0008}{1+exp\left( \frac{Ko-15}{-1.09} \right)}$ |  | Kager et al. 2000 |
| $Na_{o}=140 mM$ | Extracellular Na^+^ Concentration |  |
| $K_{o}=3 mM$ | Extracellular K^+^ Concentration |  |
| $Na_{i}=5 mM$ | Neuronal Cytosolic Na^+^ Concentration |  |
| $K_{i}N=200 mM$ | Neuronal Cytosolic K^+^ Concentration |  |
| $m=0.0530$ | Initial Probability of Activation of Na^+^ Ion Channel | Hodgkin & Huxley, 1952 |
| $hN=0.5960$ | Initial Probability of Inactivation of Na^+^ Ion Channel | Hodgkin & Huxley, 1952 |
| $nN=0.3177$ | Initial Probability of Activation of K^+^ Ion Channel | Hodgkin & Huxley, 1952 |
| $v=-65 mV$ | Resting Membrane Potential of Neuron | Hodgkin & Huxley, 1952 |
| $Ca_{No}=0.0047 mM$ | Initial Neuronal Ca^2+^ Concentration | Lee et al. 2009 |
| $ATP=20 mM$ | Initial Neuronal ATP Concentration unless mentioned otherwise |  |
| $buffer=500$mM | Initial K^+^ Buffer Capacity of Astrocyte | Kager et al. 2000 |
| $kbuffer=0$ mM | Initial K^+^ Buffered | Kager et al. 2000 |
| $Rrel=1$ (#) | Remaining Ratio of Vesicles Releasable | Lee et al. 2009 |
| $\tau_{G}=1.2 ms$ | Time constant for clearance of synaptic glutamate | Clements et al. 1992 |
| $glu=0 mM$ | Initial Synaptic Glutamate Concentration | Bennett et al. 2008 |
| $Ica=0 ms^{-1}$ | Inflow Calcium Current as Dirac Delta Function | Kager et al. 2000 |
| $rh=2*\frac{6}{25*1000}*10 \frac{\mu Mol}{ms}$ | IP_3_ Production Rate | Bennett et al. 2008 |
| $IP_{3}=0.01\mu M$ | Initial Astrocytic IP_3_ Concentration | Bennett et al. 2008 |
| $Ca_{A}=0.05\mu M$ | Initial Astrocytic Ca^2+^ Concentration | Bennett et al. 2008 |
| $hA=\frac{K_{inh}}{Ca_{A}+K_{inh}}$ | Initial Probability of Ca^2+^ Occupying its Inhibitory Binding Site | Bennett et al. 2008 |
| $EET=0 \mu M$ | Initial Extracellular EET Concentration | Bennett et al. 2008 |
| $\delta=K_{g}*K_{deg}*\frac{IP_{3}}{rh-K_{deg}*IP_{3}}$ |  | Bennett et al. 2005 |
| $iV=68e-15*.18$ L | Interstitial Volume | Mangia et al. 2009 |
| $nV=68e-15*.495$ L | Neuronal Volume | Mangia et al. 2009 |
| $bV=68e-15*.015$ L | Basal Lamina Volume | Mangia et al. 2009 |
| $aV=68e-15*.29$ L | Astrocyte Volume | Mangia et al. 2009 |
| $eV=68e-15*.0189$ L | Endothelium Volume | Mangia et al. 2009 |
| $nKL=0.7$ |  | Mangia et al. 2009 |
| $k_{diff}=1$ |  | Mangia et al. 2009 |
| $nK=4$ |  | Mangia et al. 2009 |
| $aKL=5$ |  | Mangia et al. 2009 |
| $K=8$ |  | Mangia et al. 2009 |
| $nRoi=\frac{4.4e13}{.4}\frac{s}{m mol}$ |  | Mangia et al. 2009 |
| $nRio=\frac{3.2e13}{.4}\frac{s}{m mol}$ |  | Mangia et al. 2009 |
| $nRee=\frac{3.2e13}{.4}\frac{s}{m mol}$ |  | Mangia et al. 2009 |
| $nRoo=\frac{4.4e13}{.4}\frac{s}{m mol}$ |  | Mangia et al. 2009 |
| $aRoi=\frac{5e14}{.4}\frac{s}{m mol}$ |  | Mangia et al. 2009 |
| $aRio=\frac{3.8e14}{.4}\frac{s}{m mol}$ |  | Mangia et al. 2009 |
| $aRee=\frac{3.8e14}{.4}\frac{s}{m mol}$ |  | Mangia et al. 2009 |
| $aRoo=aRio+aRoi-aRee$ |  | Mangia et al. 2009 |
| $nRLoi=2e14\frac{s}{m mol}$ |  | Mangia et al. 2009 |
| $nRLio=1e13\frac{s}{m mol}$ |  | Mangia et al. 2009 |
| $nRLee=0.1e14\frac{s}{m mol}$ |  | Mangia et al. 2009 |
| $nRLoo=nRLio+nRLoi-nRLee$ |  | Mangia et al. 2009 |
| $aRLoi=6.6e13\frac{s}{m mol}$ |  | Mangia et al. 2009 |
| $aRLio=3.3e13\frac{s}{m mol}$ |  | Mangia et al. 2009 |
| $aRLee=1e13\frac{s}{m mol}$ |  | Mangia et al. 2009 |
| $aRLoo=aRLoi+aRLio-aRLee$ |  | Mangia et al. 2009 |
| $IG/iV=1.45$ mM | Initial Concentration of Glucose in Interstitium | Mangia et al. 2009 |
| $IL/iV=1$ mM | Initial Concentration of Lactate in Interstitium | Mangia et al. 2009 |
| $NG/nV=1.26$ mM | Initial Concentration of Glucose in Neuron | Mangia et al. 2009 |
| $NL/nV=1$ mM | Initial Concentration of Lactate in Neuron | Mangia et al. 2009 |
| $AG/aV=1.42$ mM | Initial Concentration of Glucose in Astrocyte | Mangia et al. 2009 |
| $AL/aV=0.96$ mM | Initial Concentration of Lactate in Astrocyte | Mangia et al. 2009 |
| $EG/eV=3.44$ mM | Initial Concentration of Glucose in Endothelium | Mangia et al. 2009 |
| $BG/bV=1.87$mM | Initial Concentration of Glucose in Basal Membrane | Mangia et al. 2009 |
| $EL/eV=1$mM | Initial Concentration of Lactate in Endothelium | Mangia et al. 2009 |
