# Supplementary figures and images for: A Computational Model of Neuro-Glio-Vascular Loop Interactions
Source: PLoS One. 2012 Nov 20;7(11):e48802. doi: 10.1371/journal.pone.0048802 (PMC3502400; doi:10.1371/journal.pone.0048802)

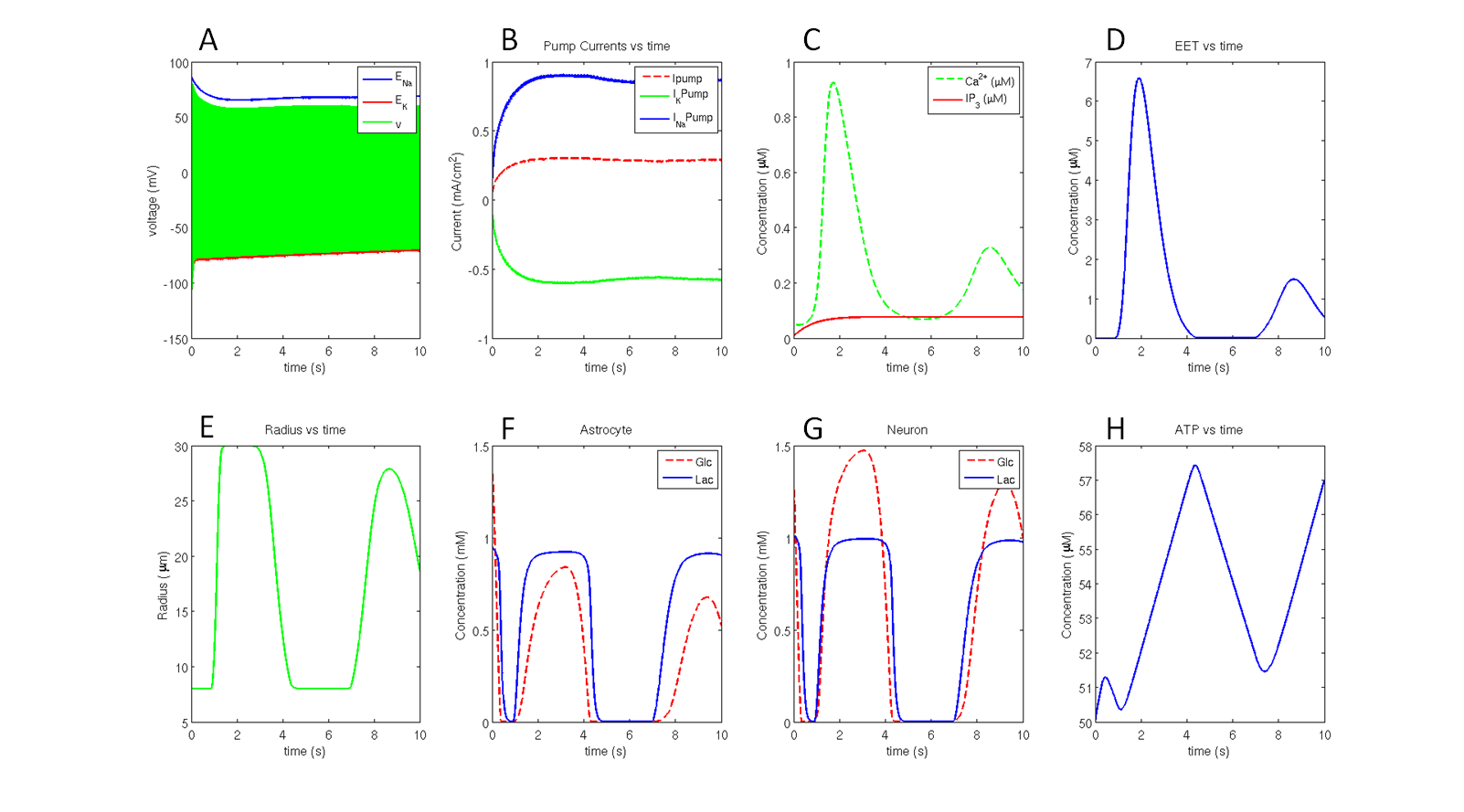

Supplement: Figure S1 — (TIF) [file pone.0048802.s003.tif]

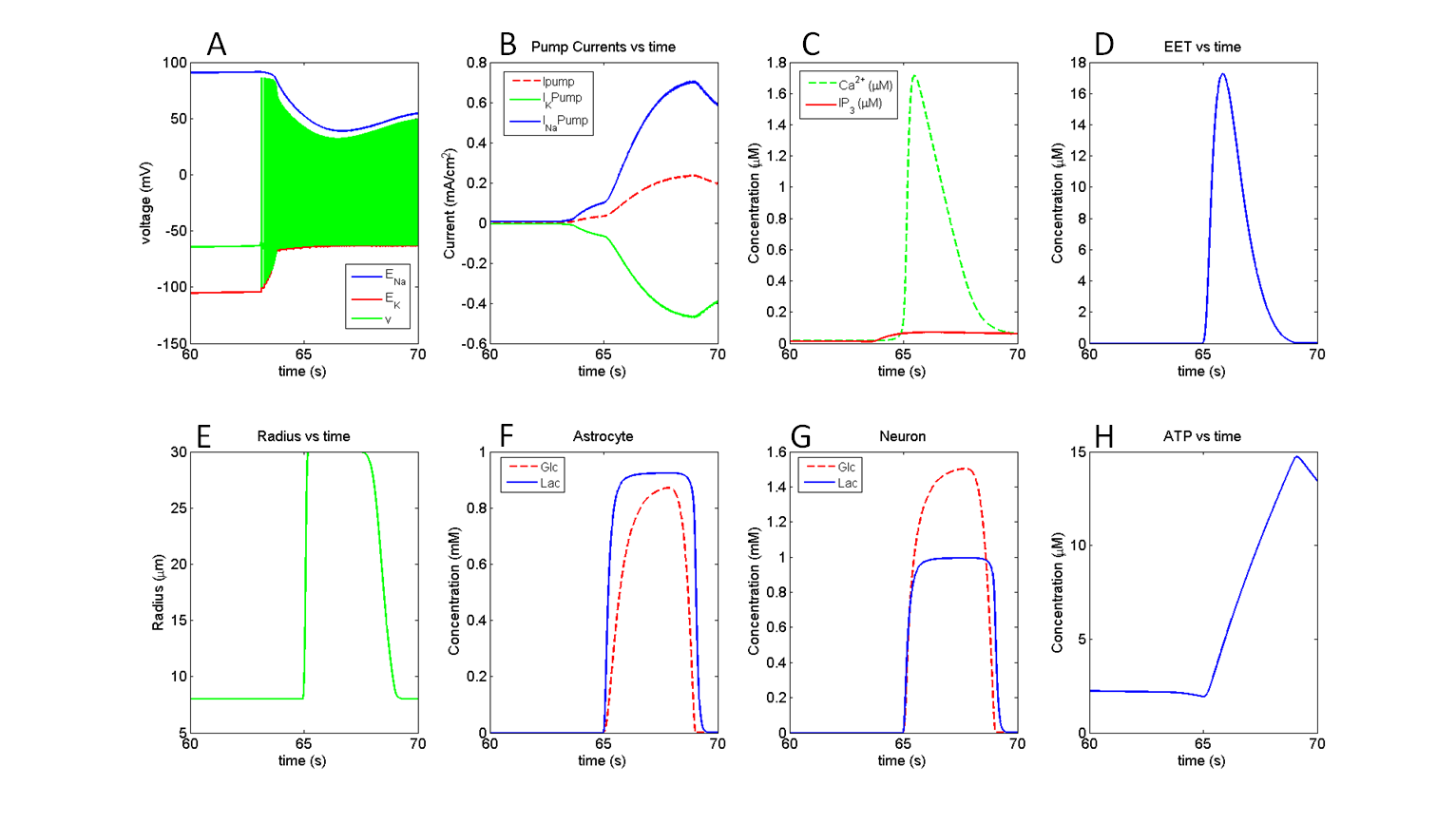

Supplement: Figure S2 — (TIF) [file pone.0048802.s004.tif]

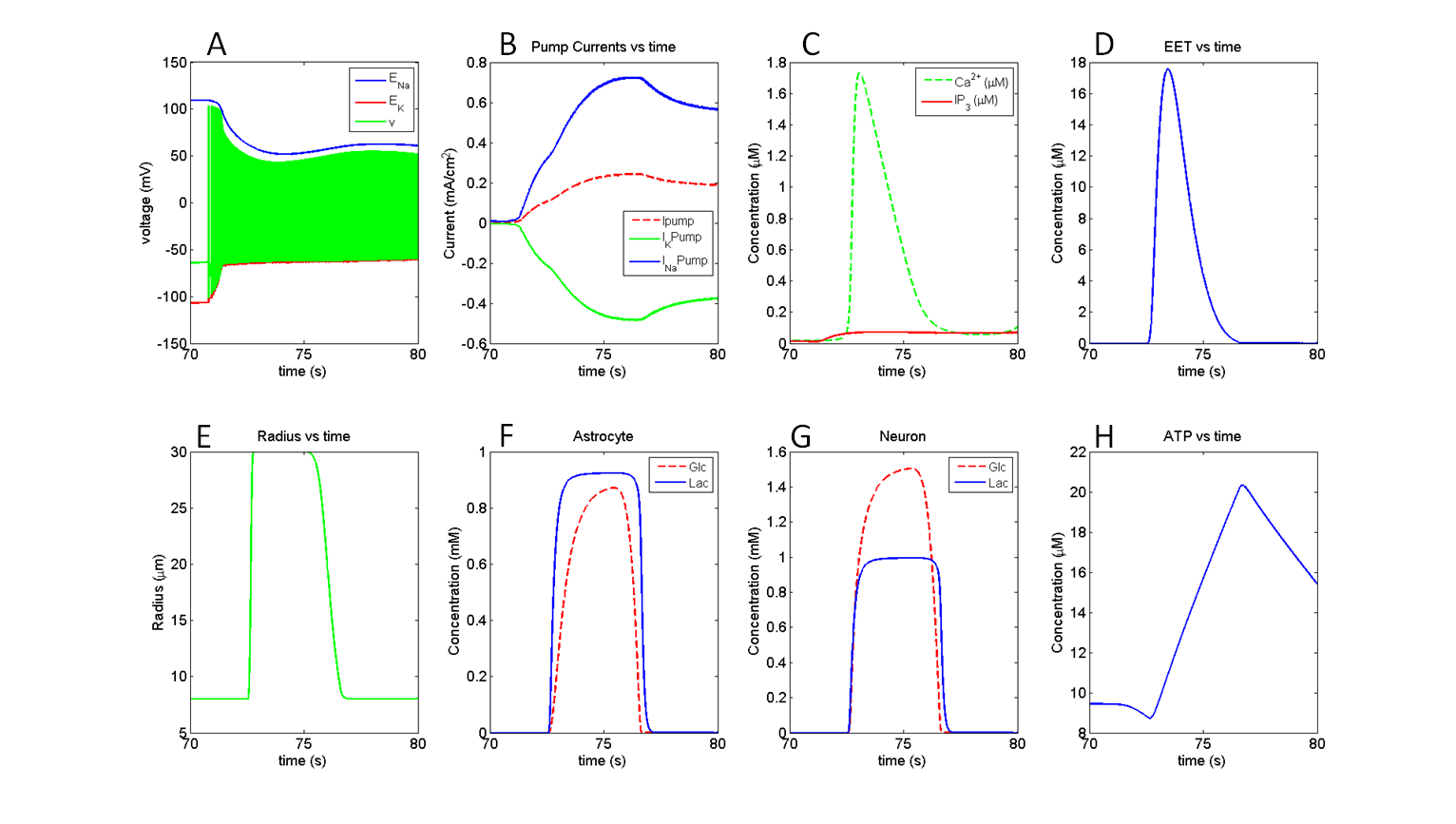

Supplement: Figure S3 — (TIF) [file pone.0048802.s005.tif]

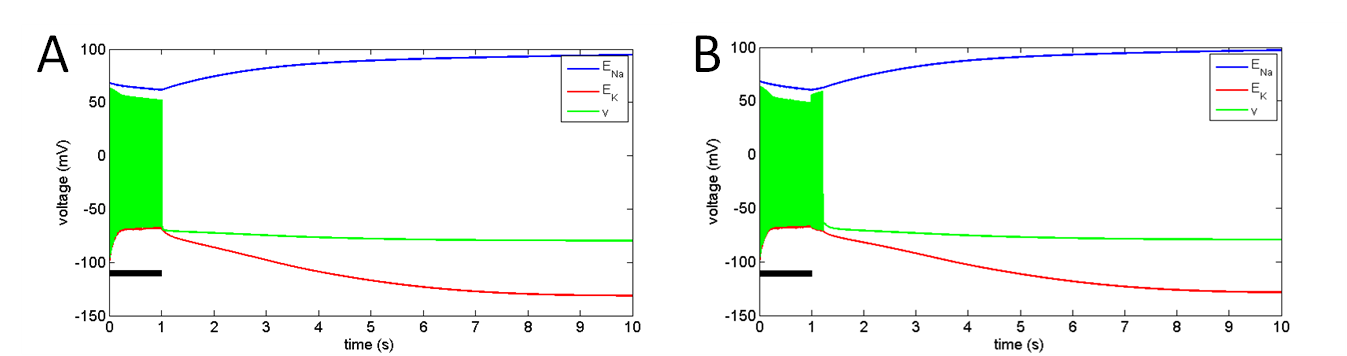

Supplement: Figure S4 — (TIF) [file pone.0048802.s006.tif]

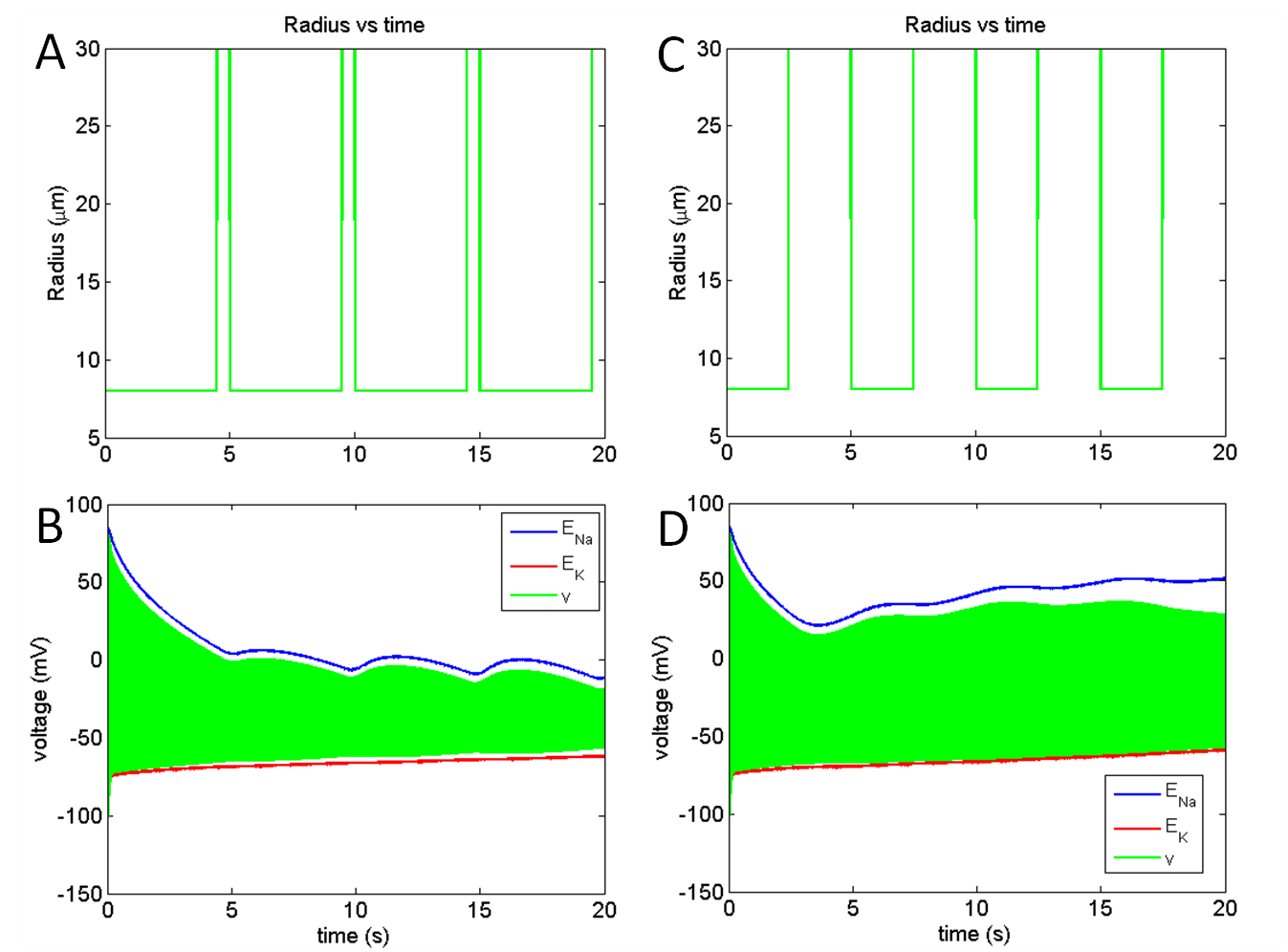

Supplement: Figure S5 — (TIF) [file pone.0048802.s007.tif]

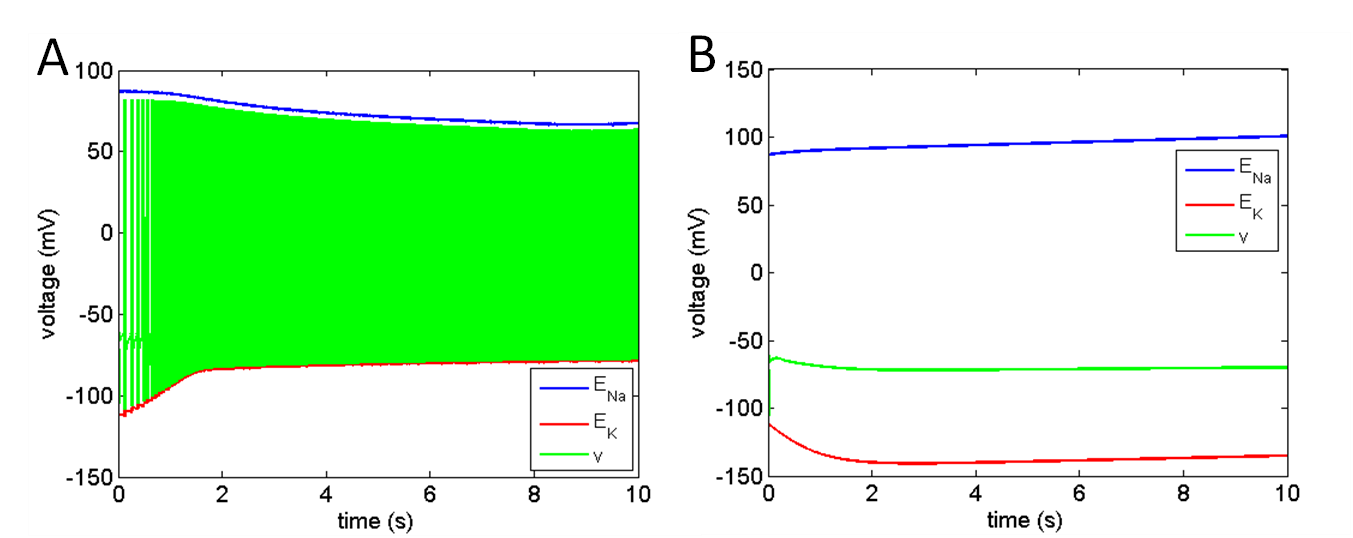

Supplement: Figure S6 — (TIF) [file pone.0048802.s008.tif]
